# *Mycobacterium tuberculosis* Sulfate Ester Dioxygenase Rv3406 Is Able to Inactivate the RCB18350 Compound

**DOI:** 10.1021/acsinfecdis.4c01030

**Published:** 2025-03-20

**Authors:** Deborah Recchia, Giovanni Stelitano, Anna Egorova, Gherard Batisti Biffignandi, Karin Savková, Radka Kafková, Stanislav Huszár, Antonio Marino Cerrato, Richard A. Slayden, Jason E. Cummings, Nicholas Whittel, Allison A. Bauman, Gregory T. Robertson, Laura Rank, Fabio Urbina, Thomas R. Lane, Sean Ekins, Olga Riabova, Elena Kazakova, Katarína Mikušová, Davide Sassera, Giulia Degiacomi, Laurent Robert Chiarelli, Vadim Makarov, Maria Rosalia Pasca

**Affiliations:** † Department of Biology and Biotechnology “Lazzaro Spallanzani,”, University of Pavia, 27100 Pavia, Italy; ‡ Fondazione IRCCS Policlinico San Matteo, 27100 Pavia, Italy; § Research Centre of Biotechnology RAS, Moscow 119071, Russia; ∥ Department of Biochemistry, Faculty of Natural Sciences, Comenius University in Bratislava, 814 99 Bratislava, Slovakia; ⊥ Mycobacteria Research Laboratories, Department of Microbiology, Immunology and Pathology, Colorado State University, Fort Collins, Colorado 80523, USA; # Colorado State University, 1682 Campus Delivery, 200 West Lake Street, Fort Collins, Colorado 80523-1782, United States; ∇ Collaborations Pharmaceuticals, Inc., Raleigh, North Carolina 27606, United States

**Keywords:** tuberculosis, antitubercular drugs, Rv3406, drug inactivation

## Abstract

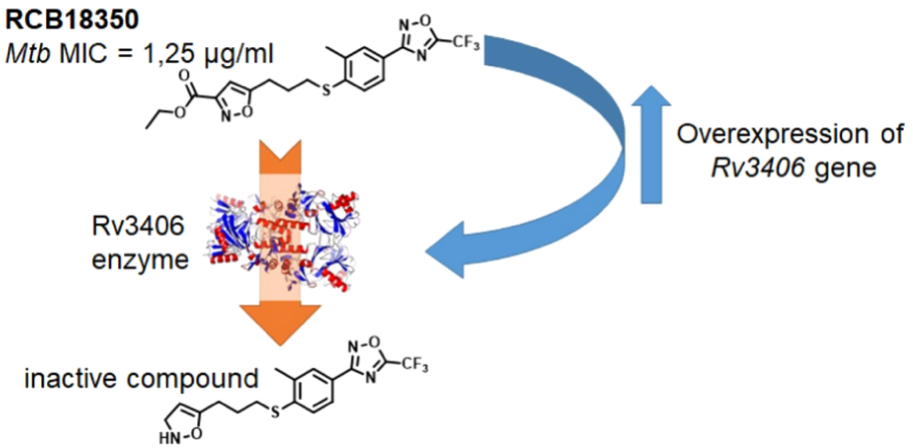

Among the critical priority pathogens listed by the World Health Organization, *Mycobacterium tuberculosis* strains resistant to rifampicin present a significant global threat. Consequently, the study of the mechanisms of resistance to new antitubercular drugs and the discovery of new effective molecules are two crucial points in tuberculosis drug discovery. In this study, we discovered a compound named RCB18350, which is active against *M. tuberculosis* growth and exhibits a minimum inhibitory concentration (MIC) of 1.25 μg/mL. It was also effective against multidrug-resistant isolates. We deeply studied the mechanism of resistance/action of RCB18350 by using several approaches. We found that Rv3406, an iron- and α-ketoglutarate-dependent sulfate ester dioxygenase, is capable of metabolizing the compound into its inactive metabolite. This finding highlights the role of this enzyme in the mechanism of resistance to RCB18350.

In 2023, tuberculosis (TB) was the world’s leading cause of death from a single infectious agent, following 3 years during which it had been replaced by COVID-19. In the same year, an estimated 8.2 million people worldwide were newly diagnosed with TB; those newly diagnosed in 2023 likely included individuals who developed TB in previous years, but whose diagnosis and treatment had been delayed due to the COVID-19 pandemic.^1^

Over the past year, efforts have been made to reestablish focus on TB, although the situation created by the pandemic has raised many concerns about future changes, increasing the possible risk of multidrug-resistant TB (MDR-TB). Indeed, drug resistance continues to be a growing threat and difficult to manage; in fact, drug-resistant forms of TB cause one in three deaths due to antimicrobial resistance.^2^

It is known that *M. tuberculosis* has intrinsic mechanisms of drug resistance, i.e., caused by physiological adaptations and permeability barriers that confer resistance to existing and newer drugs.^3^ On the other hand, in *M. tuberculosis*, the primary mechanism of acquired drug resistance is linked to mutations either in genes encoding drug targets or in antibiotic-activating enzymes. For example, *M. tuberculosis* clinical isolates resistant to bedaquiline (BDQ), which is one of the most recent drugs to enter the market, most frequently present mutations in the gene coding for the repressor of the MmpL5 efflux pump.^4^

In contrast to Gram-negative bacteria, *M. tuberculosis* presents few enzymes responsible for inactivating antitubercular drugs, such that our knowledge regarding drug inactivation by antibiotic-modifying enzymes remains limited.^5^

A noteworthy example is the β-lactamase enzyme encoded by the *M. tuberculosis* genomic *blaC* gene, which is responsible for the inactivation of β-lactams.^6^ Indeed, the deletion of the *blaC* gene has been shown to increase sensitivity to β-lactams.^7^ Furthermore, *M. tuberculosis* resistance to kanamycin could depend on its inactivation, resulting from the acetylation of the compound induced by the Eis enzyme.^8^ An additional example of drug inactivation is the acetylation of antitubercular drugs, such as isoniazid, by arylamine *N*-acetyltransferase (NAT).^9^

In 2015, Neres et al. demonstrated that Rv3406 inactivated Ty38c, an antitubercular compound belonging to the quinoxaline family and targeting the DprE1 enzyme.^10^ Rv3406 is an iron- and α-ketoglutarate (α-KG)-dependent sulfate ester dioxygenase able to decarboxylate Ty38c into its inactive keto metabolite. Specifically, Rv3406 was active in the presence of Ty38c and 2-ethylhexyl sulfate (2-EHS) (in the absence of α-KG), indicating that Rv3406 can metabolize Ty38c as a substrate instead of α-KG.^10^

Bio-AMS is a potent subnanomolar bisubstrate inhibitor of the mycobacterial biotin protein ligase, which is also inactivated by Rv3406. In this case, the enzyme oxidizes the 5′-methylene carbon of Bio-AMS to a hemiaminal, which disproportionates into biotinoyl sulfamide and adenosine 5′-aldehyde. Interestingly, four Bio-AMS analogues were designed to circumvent Rv3406-mediated chemical inactivation by blocking the oxidation through the incorporation of methyl groups at the 5′-C position of Bio-AMS.^11^

In this work, we describe a novel antitubercular **compound, RCB18350,** which is derived from the antiviral clinical candidate pleconaril, originally developed for Coxsackievirus type B,^12^ and has good activity against slow-growing mycobacteria, including *M. tuberculosis*. A multidisciplinary approach, including transcriptomic, biochemical, and microbiological procedures, was employed to elucidate its mechanism of action/resistance. Particularly, we found that the Rv3406 enzyme metabolizes **RCB18350** by producing an inactive metabolite.

## Results and Discussion

### Prediction of **RCB18350** as a Potential Anti-TB Molecule Using Machine Learning Models

**RCB18350** was initially predicted using our *M. tuberculosis* Bayesian machine learning models at 100 nM, 1 μM, and 10 μM thresholds. The molecule was predicted to be inactive in the 100 nM and 1 μM models but active in the 10 μM model (prediction score: 0.543). A comparison of **RCB18350** and a set of *M. tuberculosis* drug hits and leads used the extended connectivity circular fingerprints (ECFP6) for calculating the Tanimoto similarity. The highest Tanimoto similarity compound with the ECFP6 fingerprints had a similarity score of 0.39. When this was repeated with minimum description length (MDL) keys, the most similar molecule had a Tanimoto similarity of 0.74.

These results suggest that **RCB18350** is dissimilar to known *M. tuberculosis* drugs, hits, and leads described previously.^13^ A t-SNE plot using ECFP6 fingerprints to visualize previously curated *M. tuberculosis* drugs, hits, and leads^13^ showed **that RCB18350** was within the property space represented by these fingerprints but not particularly close to other molecules (Figure S1) which suggests it is likely unique when compared to existing TB drugs, hits, and leads.

### **RCB18350** Is Active against *M. tuberculosis*, Other Slow-Growing Mycobacterial Species, and *M. tuberculosis* Drug-Resistant Clinical Isolates

To support computational findings, a narrowly focused library of **RCB18350** derivatives and analogues was tested against *M. tuberculosis* H37Rv growth (Table S1). Almost all of these compounds were inactive against *M. tuberculosis* H37Rv, except for the analogue **RCB14148** which contains an oxygen atom instead of a sulfur atom and a 3-carbomethoxy group in the isoxazole ring (4-fold decrease in activity compared to **RCB18350**) and the derivative **RCB22135** that contains a 3-carbomethoxy group in the isoxazole ring (2-fold decrease in activity). The ethyl ester of 5-(3-((2-methyl-4-(5-(trifluoromethyl)-1,2,4-oxadiazol-3-yl)phenyl)thio)propyl)isoxazole-3-carboxylic acid (**RCB18350**) showed high activity against *M. tuberculosis* H37Rv with an MIC of 1.25 μg/mL (Table S1) and was therefore selected for extended testing against mycobacterial strains. As reported in Table 1, the *M. tuberculosis* drug-resistant isolates exhibited the same MIC as that of the wild-type strain. **RCB18350** was also active against *M. avium* and *M. bovis* BCG growth; by contrast, *M. abscessus* and *M. smegmatis* were not sensitive to this compound (MIC > 64 μg/mL) (Table 1).

**Table 1:** Activity of RCB18350 against Mycobacterial Strains^a^

	MIC (μg/mL)						
mycobacterial strains	RCB18350	STR	INH	MOX	CLT	AMK	RIF
*M. tuberculosis* H37Rv	1.25	0.25	0.03	0.06			
*M. tuberculosis* H37Rv, RpoB (S450L)	1× MIC		0.06	0.06			
*M. tuberculosis* H37Rv, GyrA (D94K)	1× MIC		0.03	>2			
*M. tuberculosis* IC1^b^ (MDR clinical isolate resistant to STR, INH, RIF, EMB, ETH)	1× MIC		>2				
*M. tuberculosis* IC2^b^ (MDR clinical isolate resistant to STR, INH, RIF, EMB, PYR, ETH, capreomycin)	1× MIC		>2				
*M. abscessus* ATCC 19977	>64				1	4	
*M. avium* 700891 (MAC 101)	4				0.06		0.06
*M. smegmatis* mc^2^155	>64						
*M. bovis* BCG	1–5						

a Abbreviations used: STR: streptomycin; INH: isoniazid; MOX: moxifloxacin; CLT: claritromycin; AMK: amikacin; RIF: rifampicin.

b MIC determination onto solid medium.

In conclusion, **RCB18350** inhibited the growth of *M. tuberculosis* drug-resistant strains, including MDR isolates, *Mycobacterium bovis* BCG, and *Mycobacterium avium*, confirming its activity against slowly growing mycobacteria. By contrast, **RCB18350** was inactive against rapidly growing mycobacteria, such as *Mycobacterium smegmatis* and *Mycobacterium abscessus* (Table 1).

The intracellular activity of **RCB18350** was also determined *ex vivo* in THP-1 macrophages infected with *M. tuberculosis* H37Rv, *M. avium*, and *M. abscessus*. This compound showed an inhibition of 86% of *M. tuberculosis* intracellular growth at MIC concentration, while it is not active against *M. abscessus* and is poorly effective against *M. avium* (Table S2). In conclusion, **RCB18350** exhibits limited intracellular activity against the slow-growing *M. tuberculosisandM. avium*, while it is not effective intracellularly against the rapidly growing mycobacterium *M. abscessus*.

### **RCB18350** Has Bacteriostatic Activity, and It Is Not Toxic

**Figure 1:**
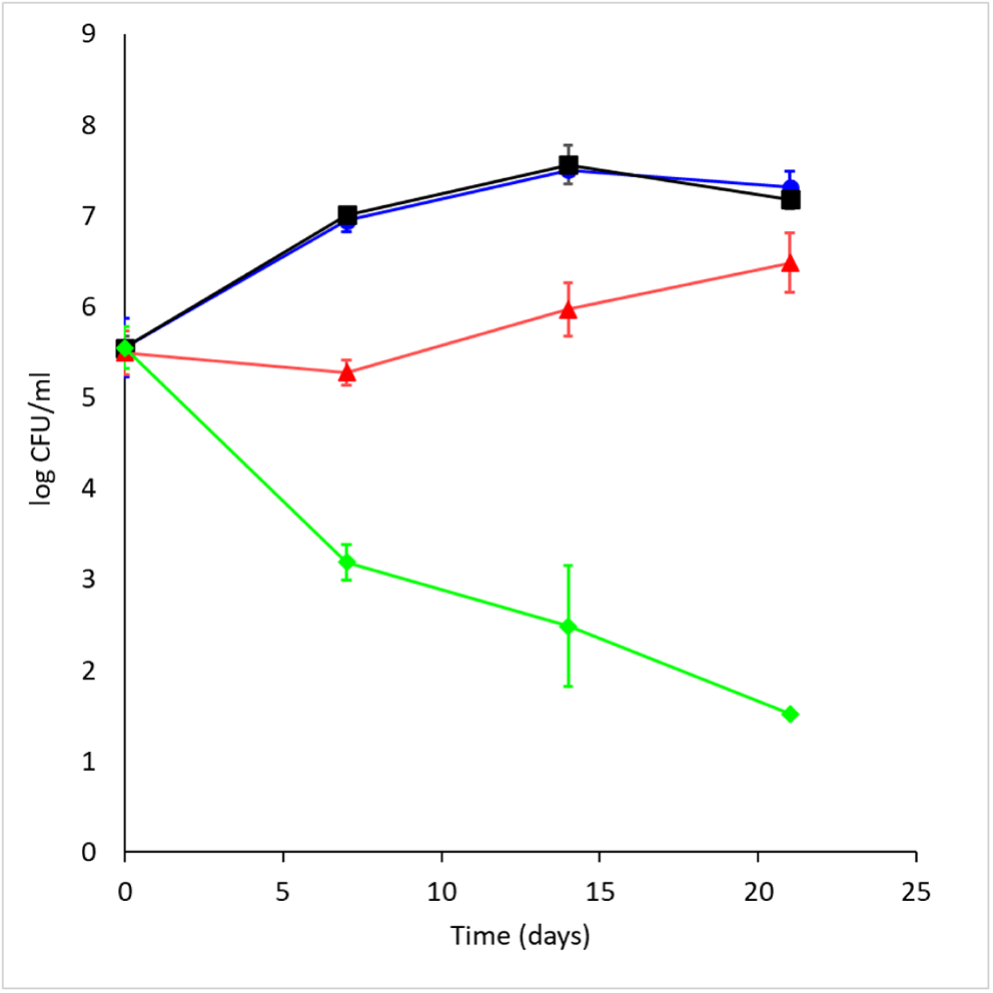
Time-killing assay of RCB18350 in *M. tuberculosis*. Blue circles: control; black squares: RCB18350 1× MIC (1.25 μg/mL); red triangles, RCB18350 40× MIC (50 μg/mL); green diamonds: moxifloxacin 10× MIC (0.6 μg/mL). Data are the mean ± standard deviation of three different experiments.

Time-kill assays (TKAs) were performed using the *M. tuberculosis* H37Rv strain and different **RCB18350** concentrations. **RCB18350** was demonstrated to have bacteriostatic activity against *M. tuberculosis* cells. Indeed, during the first 7 days of incubation, in the presence of 40× MIC of **RCB18350,** there was a slight attenuation of growth, followed by a resumption of growth from day 7 onward (Figure 1).

Cytotoxicity testing against THP-1 cells determined that **RCB18350** had an IC_50_ value >32 μg/mL and a therapeutic index (ratio of IC_50_:MIC) of 4. This compound was also nontoxic (>128 μg/mL) against HepG2 and HeLa cells. The therapeutic index for HepG2 and HeLa was ≥ 16. Mitomycin C was included as a positive control (Table S3).

### Study of the Mechanism of Action of **RCB18350**

To investigate the mechanism of action of **RCB18350**, we first attempted to generate *in vitro***RCB18350**-resistant mutants in *M. tuberculosis*. It is noteworthy that mutations in a gene encoding an essential cellular drug target confer resistance, thereby contributing to the study of the mechanism of action of antimicrobials.^14,15^ We were unable to isolate *M. tuberculosis* mutants resistant to **RCB18350**. Furthermore, attempts to use *M. bovis* BCG were similarly unsuccessful (data not shown).

Then, we screened a panel of *M. tuberculosis* mutants resistant to known drugs, harboring identified mutations in genes encoding targets (*dprE1, mmpL3, qcrA, qcrB,* and *pyrG*)^14,16−18^ and associated mechanisms of drug resistance.^19^ However, none of these strains displayed cross-resistance to RCB18350 (Table S4), suggesting that **RCB18350** could have a different mechanism of resistance/action.

Therefore, alternative strategies were considered to obtain more relevant information about the mechanism of action of **RCB18350**.

### Metabolic Labeling in the Search for the **RCB18350** Target

**Figure 2:**
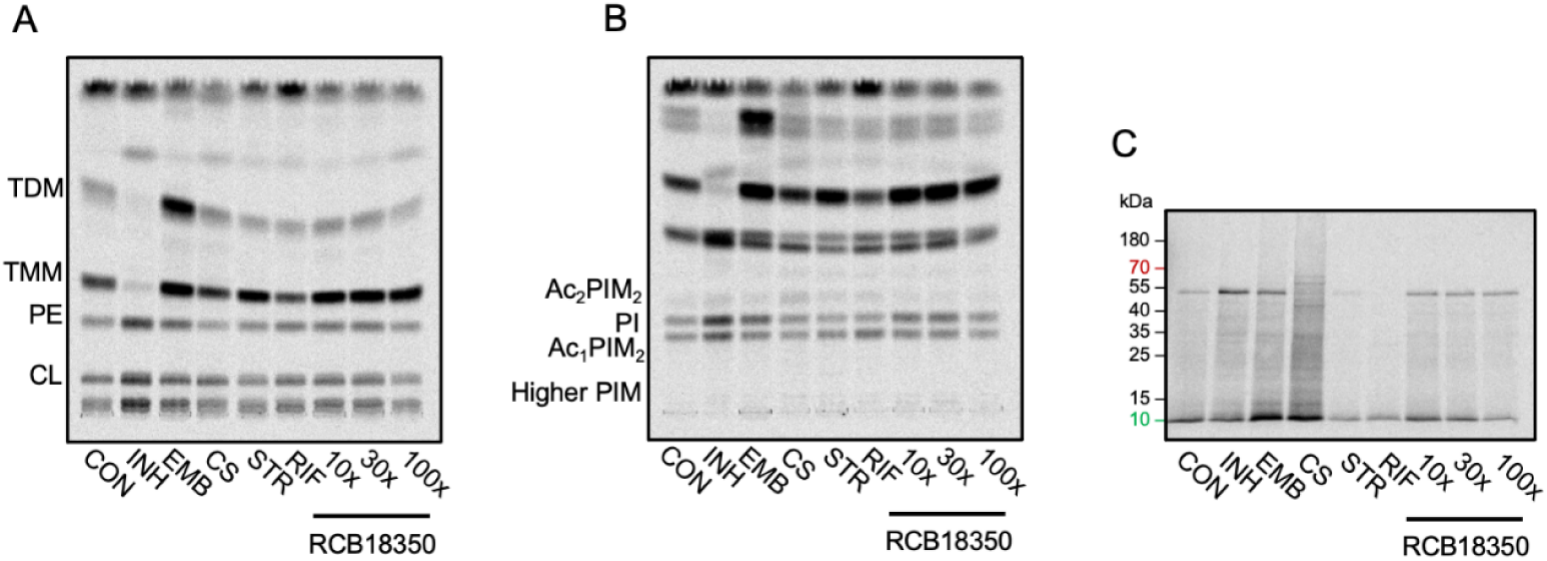
Metabolic labeling of *M. tuberculosis* H37Rv with [^14^C]-acetate (A, B) or [^14^C]-leucine (C). Lipids extracted from the [^14^C]-acetate-radiolabeled cultures grown at 10× MIC of the control drugs isoniazid (INH), ethambutol (EMB), D-cycloserine (CS), streptomycin (STR), rifampicin (RIF), and RCB18350 at 10×, 30×, and 100× MIC were analyzed by TLC in CHCl_3_/CH_3_OH/H_2_O (20:4:0.5) (A) or CHCl_3_/CH_3_OH/NH_4_OH/H_2_O (65:25:0.5:4) (B). Proteins were labeled with [^14^C]-leucine, separated by SDS-PAGE, and subsequently transferred to nitrocellulose (C). Radioactive signals were visualized by phosphorimaging. TDM, trehalose dimycolates; TMM, trehalose monomycolates; CL, cardiolipin; PE, phosphatidyl ethanolamine; PIM, phosphatidylinositol mannosides; and PI, phosphatidylinositol.

Cell wall and protein synthesis are among the most vulnerable targets of antibiotics. Consequently, we decided to examine the effects of **RCB18350** on these pathways by metabolic labeling. To monitor cell wall synthesis, we radiolabeled *M. tuberculosis* H37Rv grown in the presence of **RCB18350** and control drugs with [^14^C]-acetate. Lipids extracted from the radiolabeled bacteria were separated by TLC and visualized by phosphorimaging. The control drugs, isoniazid, targeting the synthesis of mycolic acids via InhA inhibition, or ethambutol, inhibiting the buildup of the arabinan portion of the cell wall core heteropolysaccharide arabinogalactan due to inhibition of arabinosyltransferase EmbB, show the expected profiles (i.e., a loss of trehalose monomycolates and trehalose dimycolates in the presence of isoniazid and an accumulation of trehalose monomycolates and trehalose dimycolates due to the loss of mycolate acceptor, arabinan chains) (Figure 2). Compared to these two cell wall inhibitors, the effects of D-cycloserine, targeting cytoplasmic steps of peptidoglycan synthesis (targeting d-alanine:d-alanine ligase),^20^ are not so obvious, although we could see slight quantitative changes in different forms of phosphatidylinositol mannosides. Nevertheless, the profiles of the lipids extracted from *M. tuberculosis* H37Rv grown in the presence of 10×, 30×, and 100× MIC of **RCB18350** did not allow us to make any conclusions regarding the possible cell wall target of this compound. Concurrently, we monitored protein synthesis by radiolabeling *M. tuberculosis* H37Rv cells with [^14^C]-leucine. Protein lysates were analyzed by SDS-PAGE and Western blotting, followed by phosphorimaging (Figure 2). While radiolabeled proteins are missing in the lanes corresponding to the cultures treated with streptomycin (targeting the ribosome) and rifampicin (targeting RNA polymerase), the lanes containing the samples from **RCB18350**-treated mycobacterial cells are all comparable with those of the control (without the compound). Therefore, protein synthesis does not appear to be a target of **RCB18350**.

### Transcriptomic Analysis of **RCB18350** in *M. tuberculosis*

**Figure 3:**
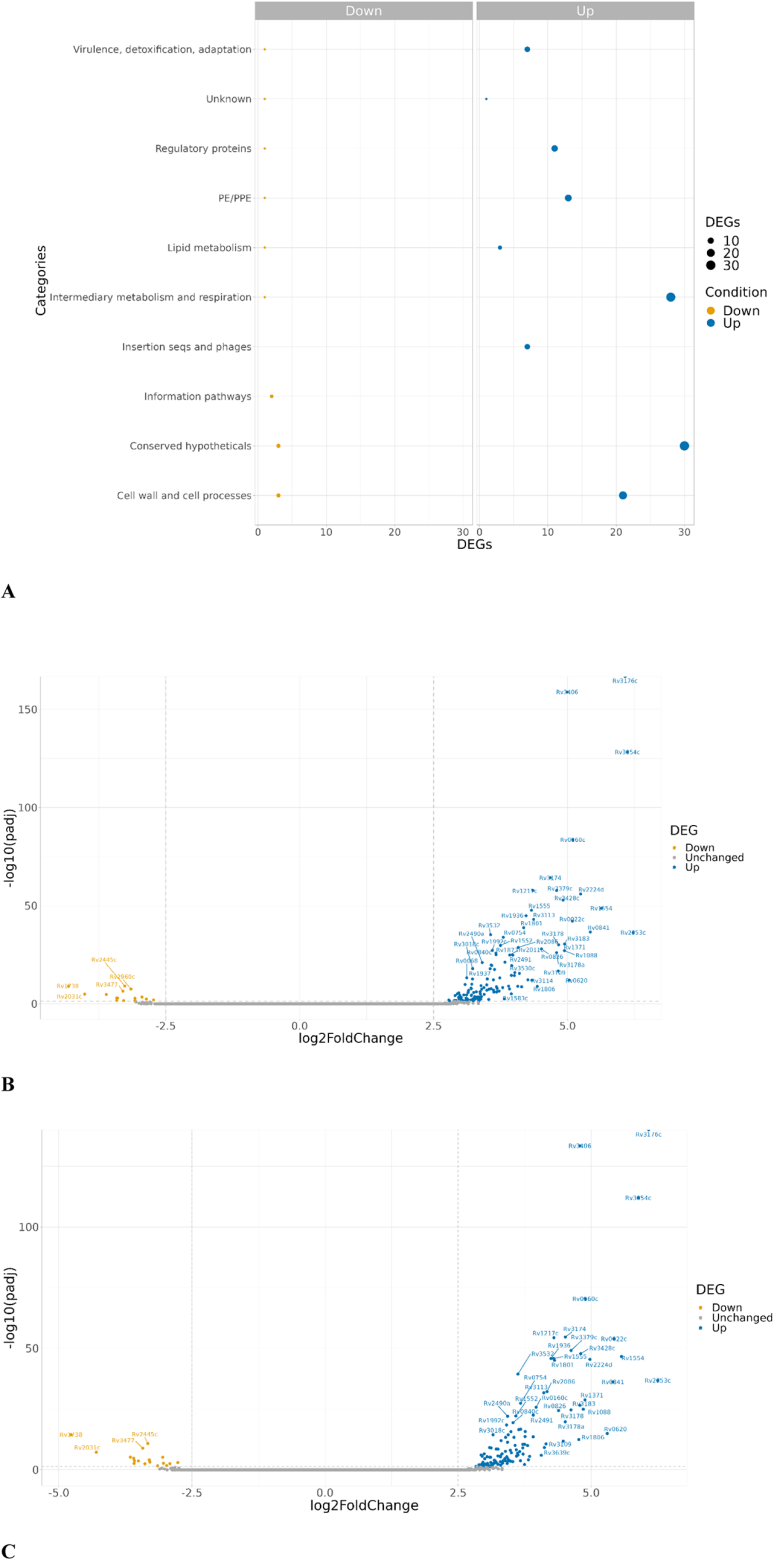
Functional characterization of upregulated/downregulated genes obtained in RNA-seq analysis, under both RCB18350 (10× – 30× MIC) treatment conditions. A. DEG Enrichment plot showing shared categories significantly upregulated and downregulated in response to RCB18350 treatment. B. Volcano plot showing *M. tuberculosis* genes up- (blue) and downregulated (yellow) in response to RCB18350 treatment (10× MIC). C. Volcano plot showing *M. tuberculosis* genes up- (blue) and downregulated (yellow) in response to RCB18350 treatment (30× MIC).

Another widely used method to investigate the mechanism of action of a compound is the characterization of the global gene expression pattern following drug treatment.^21^ Therefore, to identify differentially expressed genes (DGEs), we performed a transcriptomic analysis of *M. tuberculosis* H37Rv cells upon **RCB18350** exposure (10× MIC; 30× MIC), considering three biological replicates for each condition. Untreated samples were used as controls. Out of 3,906 coding DNA sequences, 199 (5.1%) and 191 (4.9%) genes were differentially expressed upon treatment with 10× MIC and 30× MIC of **RCB18350**, respectively, compared to the control group. In the case of the 10× MIC group, 141 genes were upregulated (2.5- to 6.07-fold), while 16 genes were under-expressed (2.5- to 4.31-fold). On the other hand, in the case of the 30× MIC group, the number of upregulated genes was 132 (2.5- to 6.2-fold), while the number of downregulated genes was 18 (2.5- to 4.7-fold). Collectively, 137 genes were found to be differentially expressed at least 2.5-fold (adjusted *p*-value < 0.05) in both treatment conditions; 123 genes were commonly upregulated in both conditions, while 14 genes were repressed (Figure 3 and Table S5). For these genes, a functional annotation analysis was performed using Mycobrowser (https://mycobrowser.epfl.ch/), based on TubercuList.^22,23^ The genes overexpressed under both conditions were found to belong to nine categories. The categories with the highest number of genes were “conserved hypotheticals”, i.e., the function of the protein is still unknown, and “intermediary metabolism and respiration”.

*The Rv3406* gene stands out as one of the most overexpressed genes in response to **RCB18350** exposure. This gene encodes an iron- and α-ketoglutarate-dependent sulfate-ester dioxygenase. Indeed, this enzyme is already known to be involved in the mechanism of resistance to different antitubercular compounds, such as 5′-[*N*-(D-biotinoyl)sulfamoyl]amino-5′-deoxyadenosine (Bio-AMS), an inhibitor of the mycobacterial biotin protein ligase (MtBPL),^11^ and the carboxyquinoxaline DprE1 inhibitors.^10^ Therefore, we hypothesized that Rv3406 could metabolize **RCB18350**.

Another interesting gene that was found to be overexpressed was *mesT*, belonging to the functional category “virulence, detoxification, adaptation”, and encoding a putative epoxide hydrolase that enables the conversion of toxic epoxides into more water-soluble and less toxic diols.^24^

Among the most upregulated genes, there were also two transcriptional regulators belonging to the WhiB family: WhiB5 and WhiB6. Genes encoding WhiB5 and WhiB6 transcriptional factors are often upregulated in *M. tuberculosis* multidrug-resistant (MDR) clinical isolates.^25^

Interestingly, several genes known to be induced by metals were upregulated after **RCB18350** treatment, such as *arsC*, *narK3*, *ctpJ*, and *ctpG* (coding for metal or cation transporters). *cyp144* and *cyp132,* coding for P450 cytochromes, are two of the more induced genes present in the intermediate metabolism and respiration categories upon treatment. Furthermore, the two overexpressed genes *frdD* and *frdC* code for two subunits of the fumarate reductase complex involved in the respiration pathway. Finally, *vapC6* and *vapC18*, coding for two toxins belonging to the toxin-antitoxin (TA) systems, were upregulated. These systems are known to help the pathogen adapt to oxidative, nitrosative, and chemical starvation, as well as multidrug tolerance.^26^

These data suggest that **RCB18350** could have a pleiotropic effect on *M. tuberculosis* cells, triggering general stress responses by affecting metal homeostasis and cell permeability. The most downregulated genes were *Rv3219* and *ndkA*, belonging to the “regulatory proteins” and “intermediary metabolism and respiration” functional categories, respectively.

*Rv3219* encodes WhiB1, an essential monomeric transcription factor containing iron–sulfur clusters of the WhiB-like family, which is widely distributed in actinobacteria. WhiB1 plays multiple roles in the regulation of cell growth and the nitric oxide stress response in *M. tuberculosis*, but its underlying mechanism remains unclear.^27^

On the other hand, the essential gene *ndkA* encodes a nucleoside diphosphate kinase.

Another essential repressed gene is *rpmH*, encoding the L34 ribosomal protein. Interestingly, *rpmG2* encoding the L33 ribosomal protein was also downregulated.

Three genes coding for ESAT-6-like proteins were found among the most-repressed (*esxK, esxO, and esxP*); interestingly, two of them belong to the region of difference RD7. *Rv2348c* belonging to the RD7 region was also downregulated, confirming this finding. Two genes, *Rv1738* and *tgs1*, coding for two vaccine candidates, were also downregulated.^28,29^ These data underline that this compound could also have an antivirulence effect.

To validate the results of the transcriptomic analysis, the expression levels of three DGEs (two induced and one repressed), *Rv3406*, *mesT*, and *ndkA*, were confirmed by qRT-PCR (Table S6).

### The Dioxygenase Rv3406 Inactivates **RCB18350**

**Figure 4:**
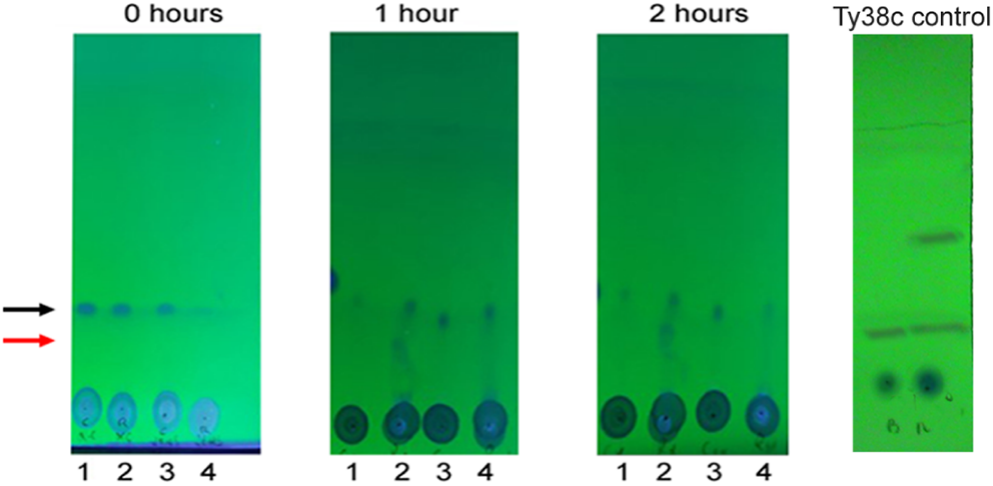
TLC analysis of the reaction products of Rv3406 at 0 (start), 1 and 2 h. Lane 1: blank control with EHS; lane 2: reaction using EHS as cosubstrate; lane 3: blank control with KG; lane 4: reaction using α-KG as cosubstrate. Black arrow points to RCB18350, and red arrow to the main metabolite. The fourth TLC corresponds to the reaction performed using Ty38C compound, a known Rv3406 substrate,^10^ as a positive control.

Encouraged by the transcriptomic analysis results, we next sought to understand the possible role of Rv3406 in **RCB18350** resistance. Consequently, to gain insight into the **RCB18350** mechanism of resistance, we first tested the sensitivity of *M. tuberculosisRv3406*-overexpressing strain to **RCB18350**. We found that this strain showed a 2-fold increase in MIC to **RCB18350**, further supporting the role of the Rv3406 dioxygenase in its resistance. An explanation for the low level of **RCB18350** resistance could be that, in this strain, the repressor coded by *Rv3405c* is functional and could partially control the expression of the *Rv3406* gene.^10^ Considering that the Rv3406 enzyme has already been found to be involved in the inactivation of other antitubercular compounds,^10,11^ we investigated this possibility for **RCB18350** as well. Thus, the Rv3406 enzyme was expressed and purified as previously reported,^10^ and its ability to metabolize **RCB18350**, using the compound as a substrate instead of α-ketoglutarate (α-KG) or ethylhexyl sulfate (EHS), was assayed. As depicted in the TLC of the reaction products in Figure 4, an additional spot appeared only in the presence of EHS used as a cosubstrate, suggesting that **RCB18350** is used by the enzyme as a substrate in place of α-KG.

**Figure 5:**
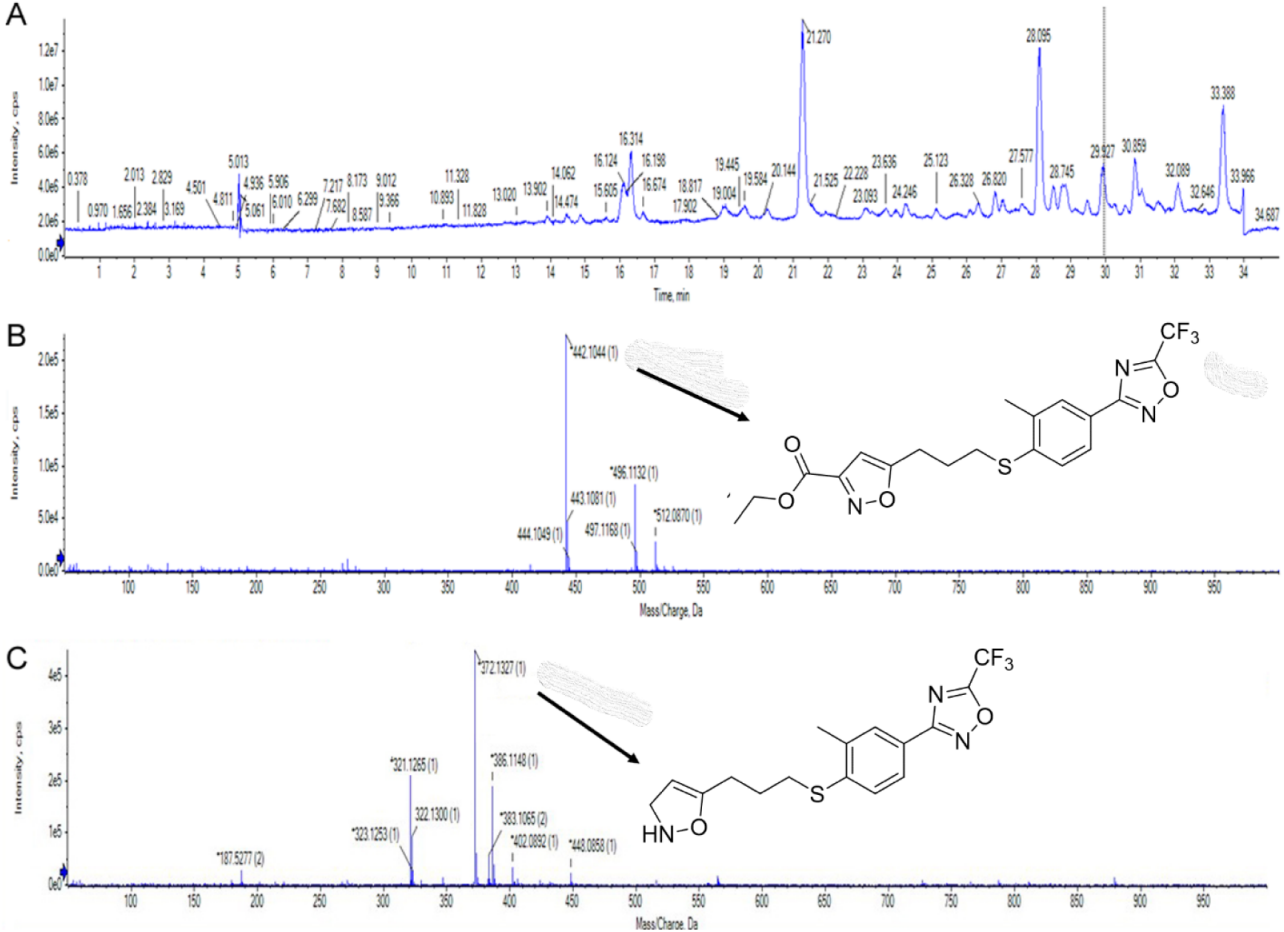
HPLC-MS analysis of the Rv3406 reaction products using RCB18350 and EHS as substrates. (A) HPLC chromatogram, monitored at 220 nm. (B) MS spectrum of the peak eluted at 29.3 min, containing unreacted RCB18350. (C) MS spectrum of the main peak eluted at 21.3 min, accountable for the decarboxylated 2-enamide derivative of RCB18350. The peak eluted at 28.1 min was also present in the negative control sample (reaction mixture without RCB18350), so was not taken into consideration.

To characterize this metabolite, we performed an HPLC -mass spectrometry analysis of the products obtained from the reaction performed with 10 mg of **RCB18350**, using 5 mg of purified Rv3406 protein and EHS as the cosubstrate, after partial purification by flash chromatography (Figure 5).

The compound eluted in the small peak at 29.3 min showed an *m*/*z* of 442.1, which is the expected **RCB18350** value, corresponding conceivably to a residual amount of unreacted compound. By contrast, the peak eluted in the main peak at 21.3 min showed an *m*/*z* of 372.1, which could account for the decarboxylated derivative of **RCB18350**. This metabolite can be achieved upon the oxidative removal of the 3-carbethoxy moiety of **RCB18350**; this reaction could be carried out by Rv3406 dioxygenase, as already shown with previous antitubercular compounds.^10,11^

Finally, the isolated metabolite was tested against *M. tuberculosis* growth, resulting in inactivity, with an MIC value >40 μg/mL, confirming that Rv3406 converts **RCB18350** into an inactive metabolite and explaining why the overexpression of this enzyme confers resistance to this compound.

Considering the mechanism of *Rv3406* inactivation, which involves the oxidative decarboxylation of the isoxazole moiety of **RCB18350**, we synthesized several derivatives with modified substituents at position 3 of the isoxazole ring: **RCB18349**, **RCB18351**, **RCB22167**, as well as sulfur-oxidized derivatives **RCB22132**, **RCB22134**, **RCB22136**, and **RCB22137**. Disappointingly, none of these substitutions were able to improve the MIC value, and a more thorough medicinal-chemistry investigation is needed.

## Conclusion

In 2024, the WHO identified 40 research priorities for antimicrobial resistance (AMR) in human health to be addressed by the year 2030, including drug-resistant pathogens causing tuberculosis.^30^ In this context, we exploited a machine learning approach, highlighting an isoxazole derivative, already studied for its antiviral activity,^31^ as a repurposed compound. Indeed, this class of isoxazole compounds is active against a panel of pleconaril-sensitive and pleconaril-resistant enteroviruses.^12^ Drug repurposing is an alternative strategy to find new applications for already characterized drugs, with the potential to save time and resources in the drug discovery process.^32^ As an example of this approach, the antiparasitic selamectin was found to be a multitarget antimycobacterial compound that kills *M. tuberculosis*.^33^

Among the identified isoxazole compounds active against *M. tuberculosis*, the best hit, **RCB18350**, is active *in vitro* against *M. tuberculosis* and *M. avium*, two slow-growing mycobacteria, including drug-resistant *M. tuberculosis* strains and MDR isolates. By contrast, the compound is not effective against the rapidly growing *M. abscessus*.

Several approaches were used to elucidate the mechanism of action of **RCB18350,** such as isolation of spontaneous resistant mutants and metabolic radiolabeling. Finally, we used transcriptomic analysis starting *M. tuberculosis* cultures treated with different concentrations of **RCB18350**. This strategy may indeed contribute to investigating the mechanism of action/resistance of antimicrobial compounds,^21^ as confirmed by previously successful studies on the mechanism of action of other new antimycobacterial compounds.^34,35^

Among the repressed genes, we found those coding for ESAT-6-like proteins and two vaccine candidates.^28,29,36,37^ These data underline that this compound could also have an antivirulence effect.

We found that **RCB18350** treatment affects both metal homeostasis and the cytoplasmic redox potential in *M. tuberculosis* cells. Among the induced genes, we found those encoding several proteins associated with cellular stress, such as cytochromes P450 and the VapC6 and VapC18 toxins. *M. tuberculosis* has multiple toxin-antitoxin systems that are involved in regulating adaptive responses to stresses associated with the host environment and drug treatment.^26^ This analysis highlighted *Rv3406* as one of the most upregulated genes upon **RCB18350** treatment, suggesting the possible involvement of the encoded enzyme in the mechanism of resistance to this compound. It has already been shown that Rv3406 is an iron- and α-KG-dependent sulfate ester dioxygenase capable of inactivating certain antitubercular compounds, such as Ty38c and Bio-AMS.^10,11^

In contrast to well-known mechanisms of resistance, such as β-lactamases,^38^ Rv3406 is still poorly studied. It should be considered in studies of new drugs when its overexpression is highlighted by transcriptomic analysis through RNA-seq and RT-qPCR and eventually in reworking and refining even current antitubercular therapies by designing new inhibitors of this enzyme. Moreover, our study demonstrated that Rv3406 is also able to metabolize **RCB18350**, using the compound as a substrate in place of α-KG. The metabolite produced by the enzymatic transformation of **RCB18350** was purified and characterized, resulting in it not being active against *M. tuberculosis* growth. This finding confirms that, also in this last case, the Rv3406 enzyme was able to inactivate a compound with antitubercular activity.

Finally, TKA was performed, highlighting that **RCB18350** has bacteriostatic activity against *M. tuberculosis*. Interestingly, the mechanism of **RCB18350** inactivation, through an enzyme whose expression is upregulated by the compound itself, could explain the peculiar behavior of this time-killing assay. Conceivably, after the first 7 days of incubation, during which the compound exerted bacteriostatic activity, the overexpression of *Rv3406* led to an increase in the amount of the enzyme suitable to inactivate **RCB18350**, allowing subsequent regrowth of the bacterium (Figure 1).

As shown in Table S1, a wide range of pleconaril derivatives were tested against *M. tuberculosis* growth, including those with different substituents at the 3-position of the oxazole, but only compounds with a COOAlk substituent were active (RCB14148, RCB22135), and ethyl derivatives showed better activity than their methyl analogues, which seems logical considering the established mechanism of inactivation. Consequently, for further studies, the most promising strategy to prevent enzymatic inactivation of the compound is to increase the alkyl size and obtain branched alkyls in the carboxyl group.

Overall, our findings confirm that the decarboxylating activity of Rv3406 should be taken into consideration for other potential antitubercular drugs with carboxyl groups, which might be inactivated in a similar way.

## Materials and Methods

### Chemistry

See Supporting Information.

### Culture Conditions and Bacterial Strains

Mycobacterial strains were grown at 37 °C in Middlebrook 7H9 broth or on Middlebrook 7H10 (Difco, Becton Dickinson) supplemented with 0.2% glycerol (Sigma-Aldrich), 0.05% Tween 80 (Sigma-Aldrich), and 10% OADC Middlebrook enrichment (Difco, Becton Dickinson).

### Minimum Inhibitory Concentration (MIC) Determination

The drug susceptibility of *M. tuberculosis* strains (and slow-growing mycobacteria) was determined by a resazurin-based microtiter assay (REMA).^39^ Log-phase cultures were diluted to concentrations of approximately 10^5^ CFU/mL, and 100 μL of suspensions was added to a 96-well black plate (Fluoronunc, Thermo Fisher) that had been previously prepared with 100 μL of Middlebrook 7H9 (without Tween-80) in the presence of a 2-fold serial dilution of the compound. After 7 days of incubation at 37 °C, 10 μL of resazurin (0.025% w/v) was added to each well. Following 24 h of incubation at 37 °C, bacterial viability was assessed using a FluoroskanTM Microplate Fluorometer (Thermo Fisher Scientific; excitation = 544 nm, emission = 590 nm) (range for slow-growing mycobacteria: 0.5–20 μg/mL RCB18350). The value for bacterial viability was calculated as the percentage of resazurin turnover in the absence of the compound (negative internal control). Experiments were performed in duplicate at least twice.

The determination of MIC in *M. abscessus* and *M. smegmatis* strains was established by the REMA method, using the same protocol but with 1 day of incubation at 37 °C (range for slow-growing mycobacteria: 0.5–64 μg/mL RCB18350).

MIC evaluation in *M. tuberculosis* IC1 and IC2 strains (MDR clinical isolates) was performed on a solid medium. Dilutions of *M. tuberculosis* wild-type or mutant strains (about 10^5^–10^6^ CFU/mL) cultures were streaked onto Middlebrook 7H10 plates containing 2-fold serial RCB18350 dilutions and incubated at 37 °C for around 3 weeks. Isoniazid (INH) was used as a control compound.

RCB18350 and its metabolites were dissolved in DMSO (Sigma-Aldrich). Streptomycin (Duchefa Biochemie) and INH (Pharmacopoeia Reference Standard, EDQM), used as internal control compounds, were dissolved in water.

### Time-Killing Assay

*M. tuberculosis* H37Rv cultures were grown to a final cell density of about 10^6^ cells/mL, diluted to an optical density (OD_600_) of 0.06, and prepared in a final volume of 3 mL of 7H9 medium without Tween-80 in 10 mL tubes. Based on the MIC value, RCB18350 was added at the following concentrations: 1× MIC, 10× MIC, and 40× MIC. An untreated dilution was prepared as a control. The *M. tuberculosis* cultures were then incubated at 37 °C for 21 days. At each time point (0, 7, 14, and 21 days), bacterial suspensions were carefully mixed and serially diluted 10-fold in PBS1×, and 10 μL aliquots were plated in triplicate on 7H10 agar square plates. CFUs were enumerated after 14 and 21 days of incubation at 37 °C. Moxifloxacin (0.6 μg/mL, corresponding to 10× MIC) was included as a control. The experiments were performed in duplicate. To verify the stability of the compound under the test conditions, RCB18350 was incubated in the same culture medium. After 7 days of incubation at 37 °C, no degradation products were detected by TLC (data not shown).

### *Ex Vivo* Activity against Mycobacteria

The experiments were performed as previously described.^40^ Briefly, THP-1 cell lines were cultured in RPMI-1640 (ATCC 30-2001) supplemented with 100 nM phorbol 12-myristate 13-acetate (PMA). The plates were then incubated at 37 °C with 5% CO_2_ for 72 h during which, on day two, the PMA-enriched medium was replaced with fresh complete medium without PMA. The cells were infected with a multiplicity of infection (MOI) of 10 and incubated at 37 °C with 5% CO_2_ for 4 h. The cells were then washed twice with 0.2 mL of PBS, and the medium was replaced with fresh complete growth medium containing the tested compounds. The plates were sealed and incubated at 37 °C with 5% CO_2_ for 24 h. The efficacy of the tested compounds was quantified using colorimetric indicator resazurin (Sigma R7017). Following two washing steps with PBS, the medium was replaced with 7H9 medium; 20 μL of a resazurin working solution (0.8 mg/mL mixed with sterile water and Tween-80 in a 2:1:1 ratio) was added to the culture wells. The absorbance was recorded at both 570 and 600 nm (BioTek Synergy H4 plate reader) for *M. tuberculosis* H37Rv strain after 7 days of incubation and for NTM strains after 3 to 5 days of incubation. The percent growth reduction (%GR) was calculated as follows:^40^
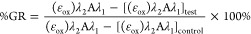
where ε_ox_ is the molar extinction coefficient of resazurin oxidized form, A is the absorbance, λ_1_ = 570 nm, λ_2_ = 600 nm, the subscript “test” refers to the test agent dilution, and the subscript “control” refers to the untreated positive growth control.

Compounds that are considered to have intracellular activity are able to inhibit >90 ± 5% of bacterial growth; intermediate activity is recorded when 50–85% of growth is inhibited compared to the untreated control.

### Cytotoxicity of **RCB18350**

Cytotoxicity was assessed in HepG2 cells (ATCC HB-8065), HeLa cells (ATCC CCL-2), and THP-1 cells (ATCC TIB-202) using the colorimetric MTT (methylthiazole tetrazolium, Sigma M6494) and resazurin (Sigma R7017) assay^41^ and as previously described.^40^ Briefly, THP-1, HepG2, and HeLa cells (1 × 10^5^ cells/mL) were cultured in 96-well plates and incubated at 37 °C with 5% CO_2_ overnight. The tested compounds were prepared as 2-fold serial dilutions and incubated under the same conditions. A volume of 200 μL was transferred from the compound plate to the corresponding wells of the tested cell plate and incubated for 24 h, including positive control (mitomycin C-treated cells, Sigma M0503). Then, 10 μL/well MTT (12 mM), 100 μL/well of detergent solution (0.1 g/mL SDS in 0.01 M HCl), and 10 μL/well of resazurin were added. After 4 h of incubation, the OD_570_ was measured using a microplate reader (BioTek Synergy H4 plate reader). The percentage cytotoxicity was calculated as follows:^40^
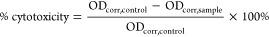
where OD_corr_ is the corrected OD.

### RNA Extraction

*M. tuberculosis* H37Rv cultures were prepared in triplicate, grown to mid-exponential phase, and then treated with two concentrations of RCB18350, corresponding to 10× and 30× MIC (12.5 and 37.5 μg/mL, respectively). Triplicates of untreated H37Rv cultures were included as controls. After 4 h of treatment, cells were pelleted, frozen in liquid nitrogen, and stored at −80 °C until use. RNA was then extracted following the protocol of the Direct-zolTM RNA Miniprep TRIzol In RNA Out kit (Zymo Research, California). The extracted RNA was then treated twice with DNase I using the TURBO DNA-free Kit (Invitrogen, Thermo Fisher Scientific, Lithuania) to remove possible traces of DNA.

### RNA-Sequencing and Analysis

RNA-Seq was performed on three biological replicates for each strain, including samples belonging to the untreated control group, 10× and 30× MIC groups, respectively. Raw reads were analyzed using FASTQC (https://qubeshub.org/resources/fastqc). After the quality check, reads were mapped onto the reference genome of *M. tuberculosis* strain H37Rv using Bowtie2.^42^ FeatureCounts software^43^ was used on the raw reads to quantify the known transcripts, considering the coding DNA sequences. Read counts were then normalized, and differential gene expression (DEG) analysis was performed through the DESeq2 R library^44^ using a Log2 Fold Change ≥ 2.5 and adjusting the *p*-value with a false discovery rate (FDR) < 0.05. Enrichment analysis of gene ontology (GO) categories (biological processes) of DEGs was performed through PANTHER.^45^ The distribution of *M. tuberculosis* genes into functional categories was also validated via the TubercuList database.^22^

### Quantitative Real Time-PCR

The purified total RNA was then reverse-transcribed into cDNA using the QuantiTect Reverse Transcription Kit (Qiagen, Germany), according to the manufacturer’s recommendations. Subsequently, quantitative real-time PCR was performed using the QuantiTect SYBR Green PCR Kit (Qiagen, Germany) and the primers listed in Table S7, following the manufacturer’s recommendations.

### Metabolic Labeling

*M. tuberculosis* H37Rv was grown shaking at 37 °C in 7H9 medium supplemented with 10% oleic acid-albumin-dextrose-catalase and 0.05% Tyloxapol until OD_600_ 0.2. Culture aliquots of 95 μL were added to Eppendorf tubes containing the drugs in 2 μL of DMSO to achieve the required final concentrations in the cultures (10× MIC for the control drugs: isoniazid, 0.5 μg/mL; ethambutol, 20 μg/mL; D-cycloserine, 23 μg/mL; streptomycin, 5 μg/mL; rifampicin, 0.3 μg/mL; and 10×, 30×, or 100× MIC for RCB18350 – 12.5 μg/mL, 37.5 μg/mL, or 125 μg/mL) and 5 μL of the radiolabel solution in water, containing 0.05 μCi of [^14^C]-acetate [specific activity: 110 mCi/mmol, American Radiolabeled Chemicals, Inc.] or 0.05 μCi of [^14^C]-l-leucine [specific activity: 328 mCi/mmol, Hartmann Analytic]. Radiolabeling was stopped after 25 h of static incubation at 37 °C. [^14^C]-acetate-labeled bacteria were used for lipid extraction. The whole cultures were transferred into 1.5 mL of CHCl_3_/CH_3_OH (2:1) and incubated for 3 h at 65 °C. The samples were then subjected to biphasic Folch wash (2×). Dried organic phases were dissolved in 50 μL of CHCl_3_/CH_3_OH/NH_3_/H_2_O (6.5:2.5:0.05:0.36). 10% of sample was subjected to TLC analysis on TLC Silica Gel F_254_ plates (Merck) in CHCl_3_/CH_3_OH/H_2_O (20:4:0.5) or CHCl_3_/CH_3_OH/NH_4_OH/H_2_O (65:25:0.5:4). Lipids were visualized by phosphor imaging (exposure time: 3 days). Cultures radiolabeled with [^14^C]-l-leucine were diluted with 900 μL of 7H9 medium and subjected to centrifugation at 14,000 g and 4 °C for 15 min. The supernatant (900 μL) was removed, and the procedure was repeated. Afterward, the cultures were autoclaved. Twenty μL aliquots of the resulting suspensions were mixed with the sample buffer, heated for 3 min at 95 °C, and centrifuged for 3 min 14,000 g. Supernatants were loaded onto 12% SDS-polyacrylamide gel. Proteins were transferred to a nitrocellulose membrane and visualized by phosphor imaging (exposure time: 7 days).

### Overexpressor *Mtb* pSODIT/*Rv3406* Strain

*The Rv3406* gene was previously cloned into the pSODIT-2 vector, as already described, obtaining pSODIT-2/*Rv3406* recombinant plasmid.^10^ The pSODIT-2/*Rv3406* recombinant vector was transformed into *M. tuberculosis* cells to overexpress the gene. To confirm the presence of the hygromycin cassette in the plasmid, the transformant colonies were screened by colony PCR using GoTaq G2 DNA Polymerase (Promega) and the primers listed in Table S7.

### Isolation of *M. tuberculosis* Spontaneous Mutants Resistant to RCB18350

The isolation of *M. tuberculosis***RCB18350**-resistant mutants was attempted by plating approximately 10^8^–10^9^ CFU from exponential growth phase cultures of H37Rv onto solid medium containing the drug at different concentrations (5×, 10×, 20× MIC). Following 6–8 weeks of incubation, unfortunately, no resistant colonies were found. The same procedure was also performed starting with *M. bovis* BCG cultures treated with the compound.

### Characterization of the Rv3406 Metabolites of RCB18350

The *M. tuberculosis* alkyl sulfatase Rv3406 was produced in recombinant form in *E. coli* BL21 (DE3) cells and purified as previously reported.^10^ Rv3406 enzymatic activity toward RCB18350 was assessed in a final volume of 100 μL of 20 mM imidazole buffer, pH 7.0, containing 200 μM FeSO_4_, 300 μM sodium ascorbate, 1–5 μM Rv3406, and 100 μM RCB18350. The reaction was started by adding 100 μM α-ketoglutarate or 2-ethylhexyl sulfate (2-EHS) and incubated at 37 °C with shaking for up to 2 h. For the blank control, no cosubstrate was added. The formation of products was monitored at intervals by silica gel thin-layer chromatography (TLC) F_254_ plates (Merck) with hexane/ethyl acetate (85:15), visualized under UV light at 254 nm, and stained with copper sulfate (10% copper sulfate and 10% phosphoric acid solution). The TLC for the control reaction with the Ty38c compound as substrate was performed with hexane/ethyl acetate (6:4).

For the isolation of the metabolites, 10 mg of RCB18350 was reacted with 5 mg of Rv3406 in a final volume of 25 mL of 20 mM imidazole buffer at pH 7.0, containing 200 μM FeSO4, 300 μM sodium ascorbate, and 100 μM 2-EHS. After 4 h of incubation at 37 °C, the reaction mixture was extracted three times with dichloromethane, and the organic phases were combined and dried under vacuum. The residue was separated by flash column chromatography (Merck SiO_2_ 60, 230–400 mesh) using hexane/ethyl acetate in an 85:15 ratio. The products were subjected to HPLC-MS analysis with a JASCO X-LC system coupled with a Thermo Fisher LTQ XL HESI-MS/MS system. The runs were monitored by measuring absorbance at 220 nm, and spectra were recorded in positive ESI resolution mode.

### Cheminformatics

Learning models for *M. tuberculosis* were generated as described previously with Assay Central software^46^ and used to predict RCB18350. The Tanimoto similarity of this molecule to molecules in this model was also compared using ECFP6 and MDL fingerprints with Discovery Studio (Biovia, San Diego).

t-SNE visualization: t-SNE^47^ embeds the data into a lower-dimensional space for visualization. 1024 ECFP6 fingerprints were generated for *M. tuberculosis* hits and leads from an earlier paper^13^ as well as RCB18350. The 1024-bit fingerprints were then embedded into a two-dimensional vector using t-SNE. All t-SNE values were generated using the scikit-learn library in Python with default hyperparameters (n_components = 2, perplexity = 30, early exaggeration = 12.0, learning rate = 200, n_iter = 1000).

## Supporting Information Available

Activity of RCB18350 derivatives and analogues against *M. tuberculosis* growth; intracellular activity of RCB18350 in *M. tuberculosis*, *M. abscessus*, and *M. avium*; cytotoxicity of RCB18350; activity of RCB18350 against a panel of characterized *M. tuberculosis* mutants; list of common differentially expressed genes (DGEs) in both RCB18350 treatments; evaluation of expression levels of three DGEs by qPCR; oligonucleotides used in this work; t-SNE plot of known TB drug hits and RCB18350; general chemistry (PDF)
