# Supplementary material for: Mycobacterium tuberculosis Sulfate Ester Dioxygenase Rv3406 Is Able to Inactivate the RCB18350 Compound
Source: ACS Infect Dis. 2025 Mar 20;11(4):986–97. doi: 10.1021/acsinfecdis.4c01030 (PMC11998004; doi:10.1021/acsinfecdis.4c01030)

SUPPLEMENTARY INFORMATION

Title: *Mycobacterium tuberculosis* sulfate ester dioxygenase Rv3406 is able to inactivate RCB18350 compound

Deborah Recchia, Giovanni Stelitano, Anna Egorova, Gherard Batisti Biffignandi, Karin Savková, Radka Kafková, Stanislav Huszár, Antonio Marino Cerrato, Richard A. Slayden, Jason E. Cummings, Nicholas Whittel, Allison A. Bauman, Gregory T. Robertson, Laura Rank, Fabio Urbina, Thomas R. Lane, Sean Ekins, Olga Riabova, Elena Kazakova, Katarína Mikušová, Davide Sassera, Giulia Degiacomi, Laurent R. Chiarelli, Vadim Makarov*, and Maria Rosalia Pasca*

Corresponding Authors

Maria Rosalia Pasca - *Department of Biology and Biotechnology "Lazzaro Spallanzani," University of Pavia, Pavia, Italy; Fondazione IRCCS Policlinico San Matteo, Pavia, Italy; Email: mariarosalia.pasca@unipv.it.*

Vadim Makarov - *Research Centre of Biotechnology RAS, 119071 Moscow, Russia; Email: makarov@inbi.ras.ru.*

Table S1. Activity of RCB18350 derivatives and analogues against *M. tuberculosis* growth.

Compounds	Structure	*M. tuberculosis* H37Rv MIC (µg/mL)	References
RCB18350	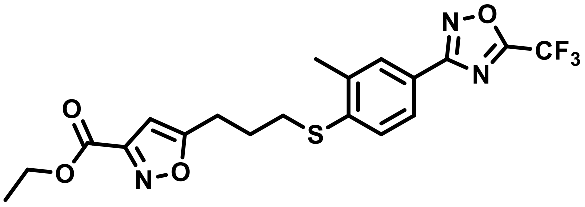	1.25	12
RCB13130	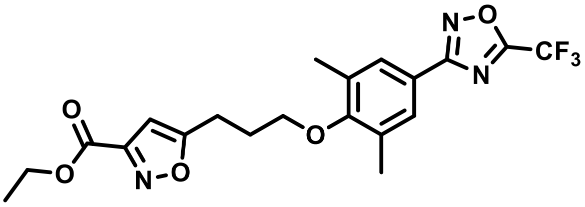	10	28
RCB14148	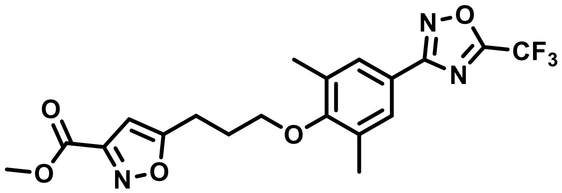	5	28
RCB14158	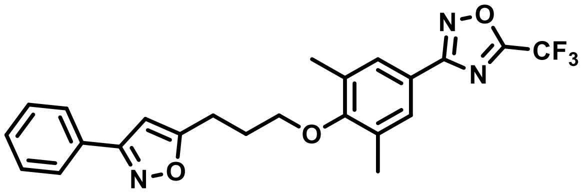	>40	28
RCB15092	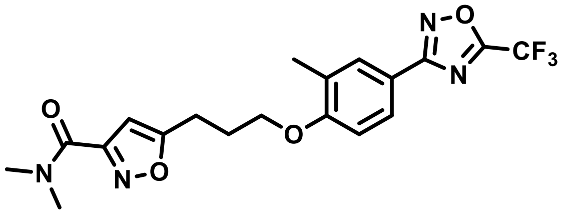	>40	28
RCB15098	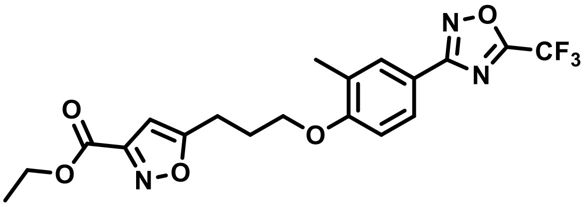	40	28
RCB18102	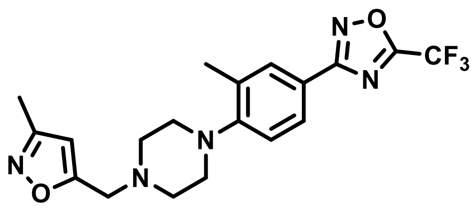	>40	12
RCB18349	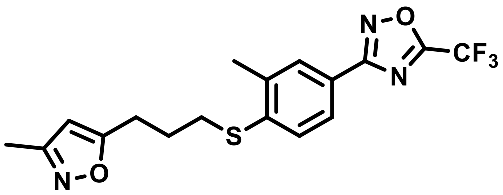	20	12

RCB18351	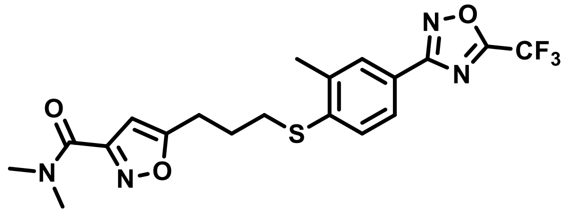	>40	12
RCB19016	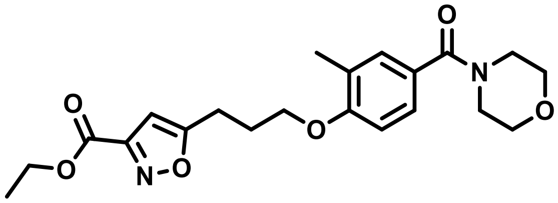	>40	This work
RCB19018	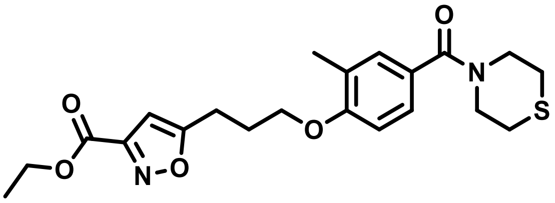	>40	This work
RCB19020	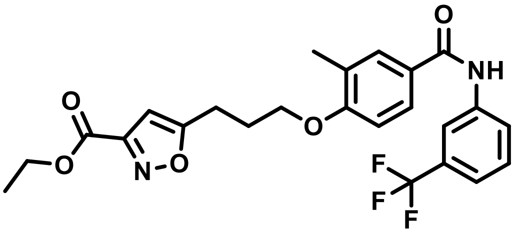	>40	This work
RCB19022	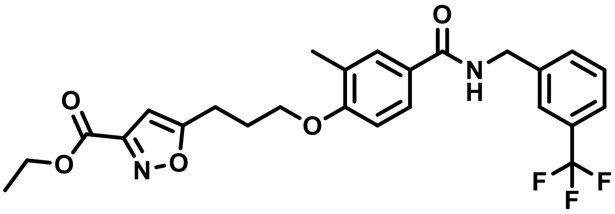	>40	This work
RCB19188	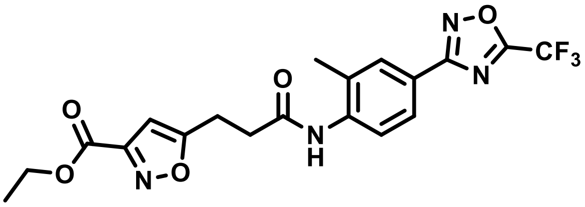	>40	12
RCB19190	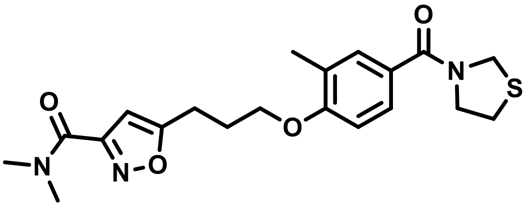	40	This work
RCB19191	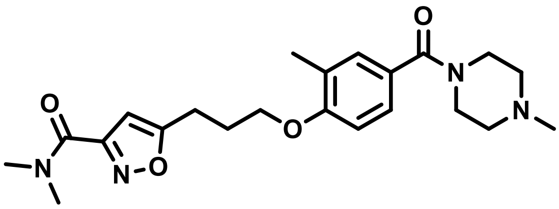	>40	This work

RCB22132	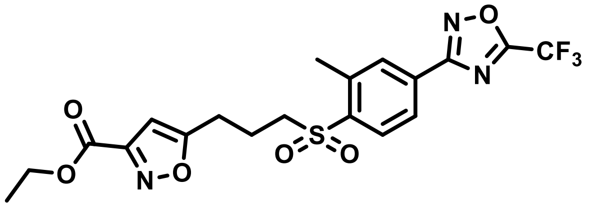	>40	This work
RCB22134	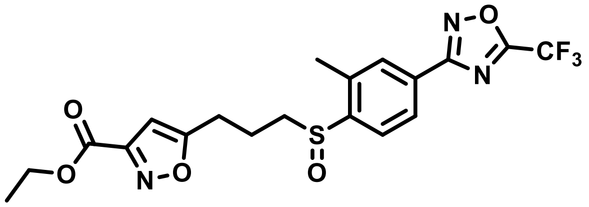	>40	This work
RCB22135	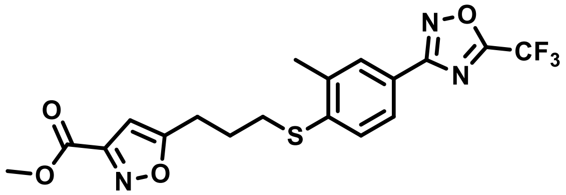	2.5	This work
RCB22136	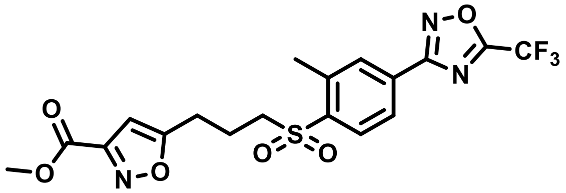	40	This work
RCB22137	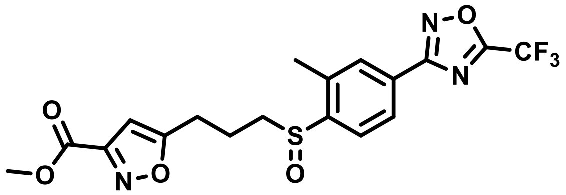	40	This work
RCB22138	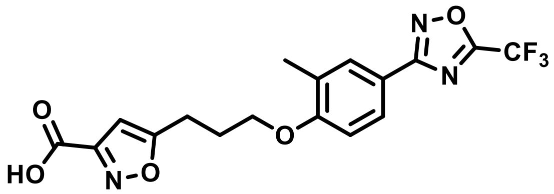	>40	This work
RCB22167	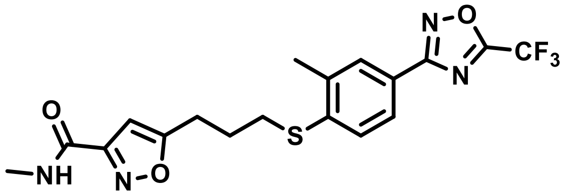	>40	This work
Streptomycin		0.25	

Table S2. Intracellular activity of RCB18350 in *M. tuberculosis*, *M. abscessus* and *M. avium*.

Compounds	***M. tuberculosis* Efficacy (% Inhibition at MIC)**	***M. avium* Efficacy (>90% Inhibition,** μg/mL**)**	***M. abscessus* Efficacy (>90% Inhibition,** μg/mL**)**
RCB18350	86	128	> 128
Clarithromycin		8	128
Rifampicin	93		
Isoniazid	91		

Table S3. Cytotoxicity of RCB18350.

Compound Name	THP-1 Cytotoxicity (IC_{50} μg/mL)	HepG2 Cytotoxicity (IC_{50} μg/mL)	HeLa Cytotoxicity (IC_{50} μg/mL)
RCB18350	37.86	>128	>128
Mitomycin C	3.722	23	10.54

Table S4. Activity of RCB18350 against a panel of characterized *M. tuberculosis* mutants.

***M. tuberculosis* strains**	**Mutation (aa change)**	**Cross-resistance(µg/mL)**	**MIC (µg/ml)**		**Reference**
			**RCB18350**	**INH**	
H37Rv			1.25	0.05	
NTB1	DprE1: C387S	BTZ043: 10 (10000X MIC)	0.5	0.05	16
DR1	MmpL3: V681I	BM212: 2.8 (4X MIC)	0.25	0.05	17
DR5	MmpL3: G253E, D46G	BM212: 2.8 (4X MIC)	0.25	0.05	17
C1	QcrA: L356V	Arylvinylpiperazine Amides: 2.5 (50X MIC)	0.5	0.05	18
B3	QrcB: G175S	Arylvinylpiperazine Amides: 2.5 (50X MIC)	0.5	0.05	18
88.7	PyrG: V186G	Thiophenecarboxamide Derivatives: 5 – 10 (5-10X MIC)	0.25	0.05	14
IC1O (MDR mutant resistant to STR, INH, ETH, RIF, EMB, BDQ)	IC1 isolate with mutation in *rv0678* (g61t) → E21Stop	Bedaquiline: 0.5 (8X MIC)	0.5	> 0.8	19

STR, streptomycin; INH, isoniazid; RIF, rifampicin; EMB, ethambutol; ETH, ethionamide; BDQ, bedaquiline; PYR, pyrazinamide; CM, capreomycin

Table S5. List of common differentially expressed genes (DGEs) in both RCB18350 treatments.

Gene	log2FoldChange	*p*-value	padj	Functional category
arsC	2,90	1,69E-06	6,52E-05	Cell wall and cell processes
uspB	2,98	8,06E-04	2,23E-02	Cell wall and cell processes
Rv2254c	3,10	8,56E-04	2,33E-02	Cell wall and cell processes
Rv3162c	3,12	7,66E-07	3,05E-05	Cell wall and cell processes
ctpJ	3,20	1,57E-10	9,12E-09	Cell wall and cell processes
Rv0488	3,20	1,51E-06	5,91E-05	Cell wall and cell processes
Rv2025c	3,21	1,36E-12	9,85E-11	Cell wall and cell processes
narK3	3,23	9,44E-05	3,08E-03	Cell wall and cell processes
Rv1686c	3,27	6,43E-06	2,44E-04	Cell wall and cell processes
mmpS5	3,27	7,83E-06	2,94E-04	Cell wall and cell processes
Rv1974	3,37	1,36E-08	6,54E-07	Cell wall and cell processes
Rv2253	3,49	2,37E-09	1,22E-07	Cell wall and cell processes
ctpG	3,60	3,91E-30	5,87E-28	Cell wall and cell processes
cut1	3,74	7,46E-16	6,19E-14	Cell wall and cell processes
Rv1735c	3,90	1,44E-11	9,55E-10	Cell wall and cell processes
Rv1735c	3,92	5,03E-10	2,76E-08	Cell wall and cell processes
Rv1217c	4,35	2,64E-61	1,72E-58	Cell wall and cell processes
Rv1371	4,95	1,87E-33	3,48E-31	Cell wall and cell processes
caeA	5,24	2,33E-59	1,14E-56	Cell wall and cell processes
Rv0841	5,42	1,07E-39	2,61E-37	Cell wall and cell processes
lppN	3,06	7,27E-07	2,92E-05	Cell wall and cell processes
Rv2044c	2,90	6,17E-04	1,77E-02	Conserved hypotheticals
Rv1929c	2,97	1,74E-05	6,12E-04	Conserved hypotheticals
Rv1044	2,97	1,21E-03	3,17E-02	Conserved hypotheticals
Rv0839	2,98	1,27E-04	4,01E-03	Conserved hypotheticals
Rv0963c	2,99	1,35E-06	5,30-05	Conserved hypotheticals
Rv1482c	3,06	1,03E-03	2,73E-02	Conserved hypotheticals
Rv0791c	3,09	2,11E-12	1,50E-10	Conserved hypotheticals
Rv0790c	3,10	4,41E-05	1,50E-03	Conserved hypotheticals
Rv3189	3,14	3,91E-08	1,75E-06	Conserved hypotheticals
Rv3471c	3,16	1,65E-03	4,13E-02	Conserved hypotheticals
Rv1804c	3,24	1,59E-03	4,00E-02	Conserved hypotheticals
Rv3188	3,26	5,95E-05	1,99E-03	Conserved hypotheticals
Rv3182	3,32	4,07E-05	1,39E-03	Conserved hypotheticals
Rv1995	3,34	8,88E-06	3,27E-04	Conserved hypotheticals
Rv2515c	3,37	1,55E-08	7,36E-07	Conserved hypotheticals
Rv2307B	3,49	3,19E-12	2,22E-10	Conserved hypotheticals
Rv1706A	3,63	2,09E-08	9,70E-07	Conserved hypotheticals
Rv1873	3,66	1,09E-28	1,47E-26	Conserved hypotheticals
Rv3529c	3,69	9,67E-09	4,71E-07	Conserved hypotheticals

Rv1499	3,72	6,38E-09	3,19E-07	Conserved hypotheticals
Rv3639c	3,95	1,93E-07	8,29E-06	Conserved hypotheticals
Rv1048c	3,95	3,80E-17	3,37E-15	Conserved hypotheticals
Rv2491	3,98	1,25E-27	1,52E-25	Conserved hypotheticals
Rv2492	4,07	1,95E-11	1,25E-09	Conserved hypotheticals
Rv2307A	4,14	3,53E-11	2,15E-09	Conserved hypotheticals
Rv3114	4,33	1,25E-14	9,24E-13	Conserved hypotheticals
Rv0826	4,51	6,98E-31	1,09E-28	Conserved hypotheticals
Rv3178	4,83	4,59E-33	8,14E-31	Conserved hypotheticals
Rv3054c	6,12	4,01E-132	5,21E-129	Conserved hypotheticals
Rv0990c	3,04	1,36E-03	3,50E-02	Conserved hypotheticals
Rv2655c	3,25	3,70E+08	1,68E-06	Insertion seqs and phages
Rv1582c	3,40	7,64E+10	4,03E-08	Insertion seqs and phages
Rv2812	3,53	2,06E-04	6,22E-03	Insertion seqs and phages
Rv3640c	3,62	7,73E-15	5,91E+13	Insertion seqs and phages
Rv1583c	3,82	9,47E-05	3,08E-03	Insertion seqs and phages
Rv2086	4,08	1,06E-31	1,72E+29	Insertion seqs and phages
Rv3428c	4,92	3,29E-56	1,42E-53	Insertion seqs and phages
Rv0331	2,88	1,11E-03	2,92E-02	Intermediary metabolism and respiration
Rv0068	3,12	6,47E-16	5,49E-14	Intermediary metabolism and respiration
Rv3175	3,12	6,80E-05	2,25E-03	Intermediary metabolism and respiration
Rv1373	3,15	2,60E-09	1,31E-07	Intermediary metabolism and respiration
Rv1937	3,23	2,96E-15	2,36E-13	Intermediary metabolism and respiration
mhpE	3,30	1,59E-08	7,49E-07	Intermediary metabolism and respiration
Rv0765c	3,32	8,20E-06	3,05E-04	Intermediary metabolism and respiration
cysD	3,39	5,01E-06	1,92E-04	Intermediary metabolism and respiration
moaD1	3,44	3,74E-07	1,55E-05	Intermediary metabolism and respiration
cyp144	3,57	1,52E-22	1,70E-20	Intermediary metabolism and respiration
moeY	3,59	2,80E-22	3,04E-20	Intermediary metabolism and respiration
pip	3,67	6,90E-28	8,96E-26	Intermediary metabolism and respiration
frdB	3,69	2,55E-10	1,46E-08	Intermediary metabolism and respiration
frdA	3,75	1,05E-32	1,78E-30	Intermediary metabolism and respiration
moaC1	3,79	8,99E-11	5,32E-09	Intermediary metabolism and respiration

Rv3530c	3,96	3,43E-22	3,61E-20	Intermediary metabolism and respiration
Rv0828c	4,02	1,36E-18	1,26E-16	Intermediary metabolism and respiration
cyp123	4,10	3,32E-18	3,01E-16	Intermediary metabolism and respiration
Rv1936	4,23	4,08E-48	1,33E-45	Intermediary metabolism and respiration
frdD	4,33	5,91E-51	2,10E-48	Intermediary metabolism and respiration
Rv3113	4,37	3,28E-46	9,84E-44	Intermediary metabolism and respiration
Rv3174	4,68	7,73E-68	6,03E-65	Intermediary metabolism and respiration
dxs2	4,80	3,26E-61	1,81E-58	Intermediary metabolism and respiration
moaA1	4,83	1,89E-19	1,79E-17	Intermediary metabolism and respiration
Rv3406	4,99	7,18E-163	1,40E-159	Intermediary metabolism and respiration
galK	5,03	1,10E-14	8,29E-13	Intermediary metabolism and respiration
Rv0560c	5,10	2,63E-87	2,57E-84	Intermediary metabolism and respiration
frdC	5,63	5,52E-52	2,15E-49	Intermediary metabolism and respiration
Rv3371	2,97	6,18E-08	2,74E-06	Lipid metabolism
echA13	3,37	2,35E-07	9,96E-06	Lipid metabolism
papA4	3,50	2,73E-15	2,22E-13	Lipid metabolism
Rv1088a	3,44	8,82E-11	5,29E-09	Not found on Mycobrowser
Rv3178a	4,80	5,94E-29	8,27E-27	Not found on Mycobrowser
PPE46	3,23	1,08E-20	1,11E-18	Pe/ppe
PE32	3,33	1,85E-04	5,63E-03	Pe/ppe
PE_PGRS43	3,41	8,30E-24	9,52E-22	Pe/ppe
PPE61	3,56	3,01E-38	6,52E-36	Pe/ppe
PE8	3,62	2,07E-09	1,08E-07	Pe/ppe
PPE34	3,67	2,97E-11	1,84E-09	Pe/ppe
PPE37	3,68	2,16E-08	9,92E-07	Pe/ppe
PE_PGRS11	3,80	6,57E-37	1,35E-34	Pe/ppe
PE4	3,83	5,32E-24	6,29E-22	Pe/ppe
PPE29	4,18	6,74E-42	1,75E-39	Pe/ppe
PE20	4,44	2,07E-11	1,30E-09	Pe/ppe
PE9	4,94	5,63E-30	8,14E-28	Pe/ppe
PPE39	6,22	2,09E-39	4,79E-37	Pe/ppe
Rv1353c	2,97	1,13E-05	4,10E-04	Regulatory proteins
Rv0324	2,99	1,13E-04	3,62E-03	Regulatory proteins
Rv2621c	3,00	3,09E-04	9,27E-03	Regulatory proteins
Rv0792c	3,20	3,48E-10	1,94E-08	Regulatory proteins
Rv3167c	3,26	1,10E-04	3,55E-03	Regulatory proteins

Rv0260c	3,35	8,68E-04	2,35E-02	Regulatory proteins
cmtR	3,55	1,33E-20	1,33E-18	Regulatory proteins
Rv2011c	3,92	1,13E-27	1,43E-25	Regulatory proteins
whiB6	4,26	7,62E-15	5,91E-13	Regulatory proteins
Rv3183	4,94	1,83E-33	3,48E-31	Regulatory proteins
whiB5	5,09	2,62E-45	7,29E-43	Regulatory proteins
Rv0326	4,01	4,83E-17	4,19E-15	Unknown
vapC6	3,21	7,06E-07	2,87E-05	Virulence, detoxification, adaptation
mce3A	3,37	1,50E-07	6,58E-06	Virulence, detoxification, adaptation
bpoA	3,39	8,03E-12	5,49E-10	Virulence, detoxification, adaptation
vapC18	3,53	7,32E-10	3,91E-08	Virulence, detoxification, adaptation
yrbE3B	3,57	3,49E-10	1,94E-08	Virulence, detoxification, adaptation
Rv3177	3,64	3,39E-20	3,31E-18	Virulence, detoxification, adaptation
mesT	6,08	0,00E+00	0,00E+00	Virulence, detoxification, adaptation
esxK	-3,28	9,16E-04	2,45E-02	Cell wall and cell processes
esxP	-3,39	3,37E-05	1,17E-03	Cell wall and cell processes
esxO	-3,61	4,06E-07	1,67-05	Cell wall and cell processes
Rv2816c	-3,07	7,08E-04	1,99E-02	Conserved hypotheticals
Rv2960c	-3,15	5,45E-10	2,95E-08	Conserved hypotheticals
Rv1738	-4,32	1,57E-11	1,02E-09	Conserved hypotheticals
rpmH	-2,73	3,47E-04	1,03E-02	Information pathways
rpmG2	-3,42	3,81E-05	1,32E-03	Information pathways
ndkA	-3,26	1,33E-11	8,95E-10	Intermediary metabolism and respiration
tgs1	-3,08	1,77E-03	4,41E-02	Lipid metabolism
PE31	-3,30	6,72E-09	3,32E-07	Pe/ppe
whiB1	-3,07	4,45E-05	1,50E-03	Regulatory proteins
Rv2348c	-3,42	9,16E-04	2,45E-02	Unknown
hspX	-4,01	2,67E-07	1,12E-05	Virulence, detoxification, adaptation

Table S6. Evaluation of expression levels of three DGEs by qPCR.

M. tuberculosis cultures	Genes					
	Rv3406		***mesT***		***ndkA***	
	Expression level	*p*-value	Expression level	*p*-value	Expression level	*p*-value
Non-treated	1.00 ± 0.19	-	1.00 ± 0.14	-	1.00 ± 0.11	-
10X RCB18350 MIC	61.96 ± 9.39	0,000357	95.74 ± 3.96	0.000874	0.21 ± 0.09	0.002982
30X RCB18350 MIC	61.05 ± 4.32	0,000018	55.26 ± 14.02	0.031801	0.22 ± 0.07	0.002128

Table S7. Oligonucleotides used in this work.

Oligonucletide name	Sequence	Purpose
RT_sigA_F	gatgacgacgaggagat	RT-qPCR of *sigA*
RT_sigA_R	gccgatctgtttgaggta	RT-qPCR of *sigA*
DR29RTRv3406_mtb_F	gcggtctccctgccca	RT-qPCR of *Rv3406*
DR30RTRv3406_mtb_R	cgtagtcatagcggttg	RT-qPCR of *Rv3406*
DR33RTRv3176c_mtb_F	gcgagcgggcgggaaac	RT-qPCR of *Rv3176c*
DR34RTRv3176c_mtb_R	gatgtgagcagcggcgtc	RT-qPCR of *Rv3176c*
DR35RTRv2445c_mtb_F	gcctcaccatcgctgc	RT-qPCR of *Rv2445c*
DR36RTRv2445c_mtb_R	atcgccgctaccaccg	RT-qPCR of *Rv2445c*
DR37RTRv1738_mtb_F	ttggcgggaaaaggaat	RT-qPCR of *Rv1738*
DR38RTRv1738_mtb_R	cggctgatgggtaaca	RT-qPCR of *Rv1738*
HYG-F	TAactagtGGAGGGGCAGTCCTCCAC	Amplification of hygromycin cassette
HYG-R	TAactagtGACCACCCTGGAGGAGATGAT	Amplification of hygromycin cassette

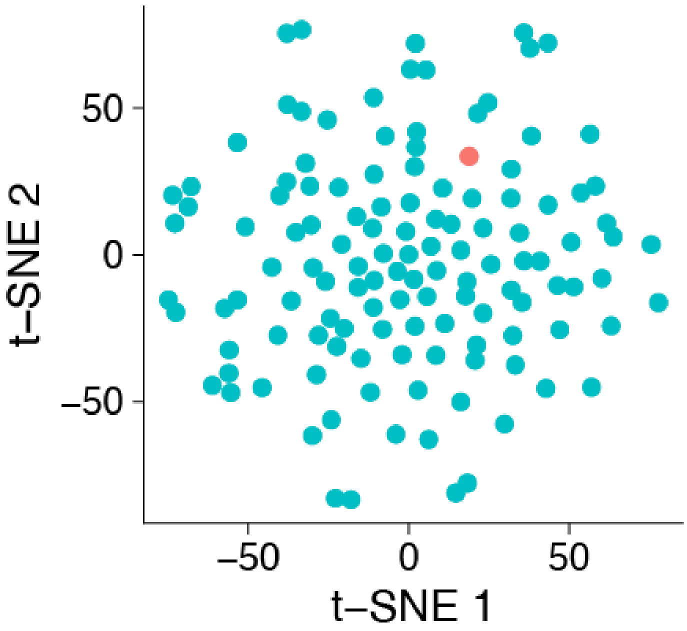

Figure S1. t-SNE plot of known TB drug hits and RCB18350. Green balls: antitubercular drugs, hits and leads; red ball: RCB18350.

General chemistry

All reagents and solvents were purchased from commercial suppliers (AlfaAesar, Acros, Chimmed) and used without further purification. The ^{1}H and ^{13}C spectra were recorded on a Bruker AC-200 (200 MHz, ^{1}H; 50 MHz, ^{13}C) NMR spectrometer. Chemical shifts were reported as ppm values values to the residual solvent peak (DMSO-d_6). Mass spectra were recorded on a Finnigan MAT INCOS 50 quadrupole mass spectrometer (EI, 70 eV) with direct injection. Purify of the final compounds was analyzed on an Agilent 1290 Infinity II HPLC system coupled to an Agilent 6460 triple-quadrupole mass spectrometer equipped with an electrospray ionization source. Chromatographic separation was performed on an Agilent Eclipse Plus C18 RRHD column (2.1 × 50 mm, 1.8 µm) at 40 ºC; sample injection volume – 0.2 µL. A mobile phase consisting of 0.1 % formic acid/water (A), and 0.1 % formic acid and 85 % acetonitrile/water (B) was programmed with gradient elution at a flow rate of 0.4 mL/min as follows: 0.0-3.0 min, 60 % B; 3.0-4.0 min, 60 % to 97 % B; 4.0-6.0 min, 97 % B; 6.0-6.1 min, 97 % to 60 % B. Mass spectrometric detection was operated in the positive ion mode. The optimal parameters were: capillary voltage 3500 V, nebulizer pressure 35 psi, gas temperature 350 ºC, gas flow rate 12 L/min. All final compounds were ≥ 95 % pure. Melting points were determined on an Electrothermal 9001 melting point apparatus (10 °C per min) and were uncorrected. Merck KGaA silica gel 60 F_{254} plates were used for analytical thin-layer chromatography. Spots were detected by a UV lamp. Yields refer to purified products and were not optimized.

Synthetic procedures and physicochemical properties for **RCB13130**, **RCB14148**, **RCB15092**, **RCB15098**, **RCB18349-18351** were previously described [12,28].

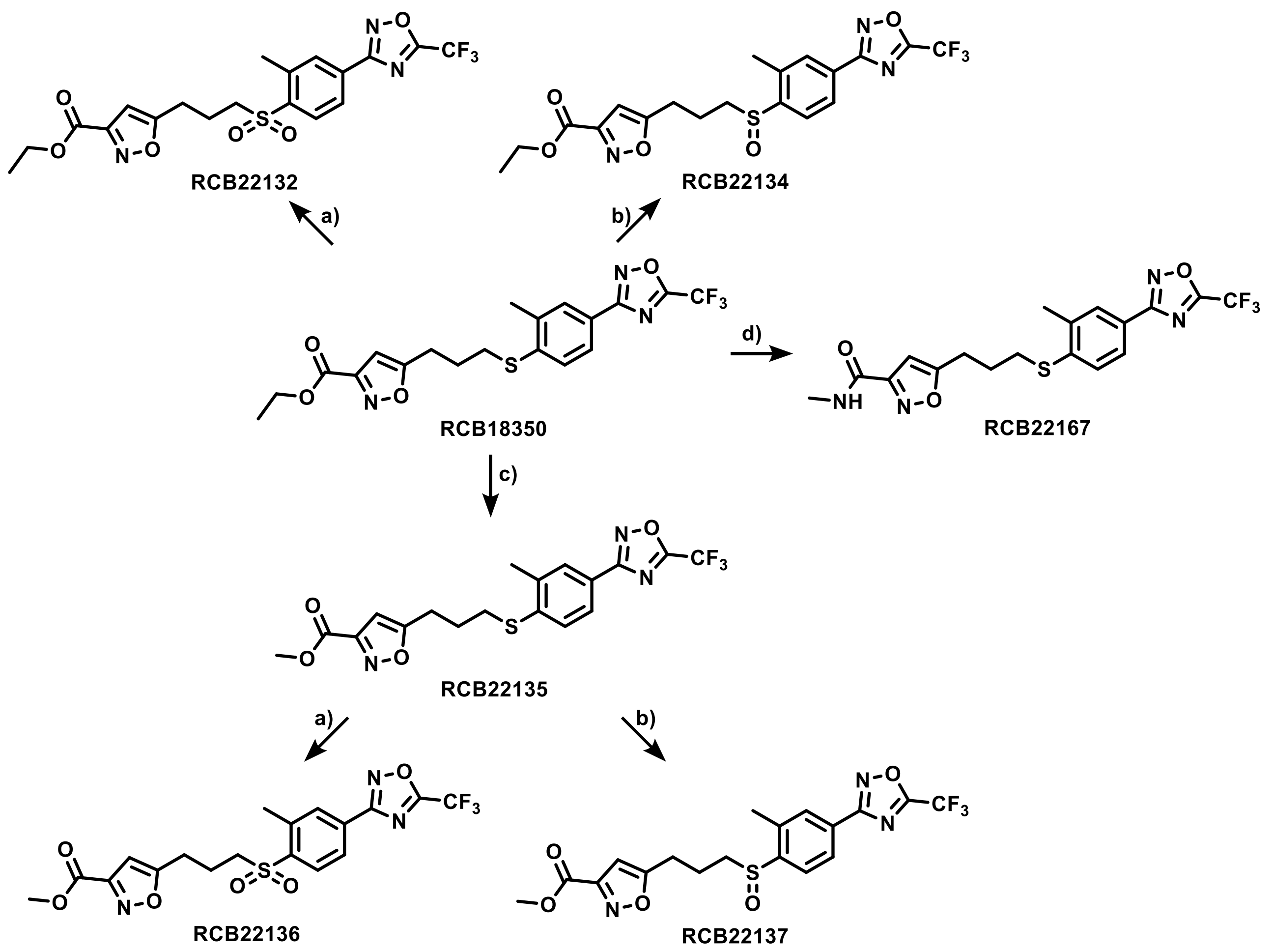

Reagents and conditions: a) 30% H_2O_2, TFAA, rt; b) 1% H_2O_2, TFAA, rt; c) MeONa, MeOH, rt; d) 40% $MeNH_{2wat}$, MeOH, rt

Scheme 1. Synthesis of RCB18350 derivatives.

Synthetic Procedures

Synthetic procedure for ethyl ester of 5-(3-((2-methyl-4-(5-(trifluoromethyl)-1,2,4-oxadiazol-3-yl)phenyl)sulfonyl)propyl)isoxazole-3- carboxylic acid RCB22132 and methyl ester of 5-(3-((2-methyl-4-(5-(trifluoromethyl)-1,2,4-oxadiazol-3-yl)phenyl)sulfonyl)propyl)isoxazole-3 - carboxylic acid RCB22136

To a solution of **RCB18350** (0.2 g) or **RCB22135** (0.2 g) in trifluoroacetic acid (1.5 mL), 30% hydrogen peroxide (0.06 mL) was carefully added, and the resulting mixture was stirred at room temperature overnight. After completion of the reaction, which was monitored by TLC, the mixture was then treated with water, the precipitate was filtered and recrystallized from methanol (**RCB22132**) or ethanol (**RCB22136**) to afford the products as white solids. **RCB22132**, Yield 89 %. M.p. 71-76 °C. MS (EI; rel. int., %): m/z 473 (M^+, 87). ^1H NMR (200 MHz, DMSO-d_6; δ, ppm): 1.30

(3H, t, J = 7.1, C$\underline{H_3}$CH$_2$O), 1.99 (2H, q, J = 7.7, CH$_2$C$\underline{H_2}$CH$_2$O), 2.74 (s, 3H, CH$_3$), 2.97 (2H, t, J = 7.5, C$\underline{H_2}$CH$_2$CH$_2$O), 3.49 (2H, t, J = 7.2, CH$_2$O), 4.34 (2H, q, J = 7.1, CH$_3$C$\underline{H_2}$O), 6.69 (1H, s, CH$_{isoxazole}$), 7.99-8.40 (3H, m, 3CH$_{ar}$). ^{13}C NMR (50 MHz, DMSO-d$_6$; δ, ppm): 13.87, 19.58, 20.31, 24.22, 52.97, 61.66, 102.08, 125.64, 129.14, 130.91, 131.37, 139.34, 140.13, 156.05, 159.39, 167.36, 174.01. **RCB22136**, Yield 78 %. M.p. 95-97 °C. MS (EI; rel. int., %): m/z 459 (M$^+$, 90). ^{1}H NMR (200 MHz, DMSO-d$_6$; δ, ppm): 1.98 (2H, p, J = 7.6, CH$_2$C$\underline{H_2}$CH$_2$O), 2.74 (3H, s, CH$_3$), 2.98 (2H, t, J = 7.5, C$\underline{H_2}$CH$_2$CH$_2$O), 3.49 (2H, t, J = 7.2, CH$_2$O), 3.88 (3H, s, COOCH$_3$), 6.71 (1H, s, CH$_{isoxazole}$), 8.15 (3H, m, 3CH$_{ar}$). ^{13}C NMR (50 MHz, DMSO-d$_6$; δ, ppm): 19.58, 20.31, 24.23, 52.65, 52.99, 102.08, 112.95, 118.36, 125.65, 129.16, 130.92, 131.37, 139.34, 140.14, 155.86, 159.87, 164.92, 165.80, 167.37, 174.07.

Synthetic procedure for ethyl ester of 5-(3-((2-methyl-4-(5-(trifluoromethyl)-1,2,4-oxadiazol-3-yl)phenyl)sulfinyl)propyl)isoxazole-3-carboxylate RCB22134 and methyl 5-(3-((2-methyl-4-(5-(trifluoromethyl)-1,2,4-oxadiazol-3-yl)phenyl)sulfinyl)propyl)isoxazole-3- carboxylic acid RCB22137

To a solution of **RCB18350** (0.1 g) or **RCB22135** (0.1 g) in trifluoroacetic acid (1 mL), 1% hydrogen peroxide (0.27 mL) was carefully added, and the resulting mixture was stirred at room temperature for 30 min. After completion of the reaction, which was monitored by TLC, the mixture was then treated with water, and the precipitate was filtered and recrystallized from methanol (**RCB22134**) or ethanol (**RCB22137**) to afford the products as white solids. **RCB22134**, Yield 86 %. M.p. 92-95 °C. MS (EI; rel. int., %): m/z 457 (M$^+$, 89). ^{1}H NMR (200 MHz, DMSO-d$_6$; δ, ppm): 1.31 (3H, t, J = 7.1, C$\underline{H_3}$CH$_2$O), 1.75-2.01 and 2.02-2.30 (1H and 1H, m, CH$_2$C$\underline{H_2}$CH$_2$O), 2.44 (3H, s, CH$_3$), 2.62-3.19 (4H, m, C$\underline{H_2}$CH$_2$C$\underline{H_2}$O), 4.35 (2H, q, J = 7.1, CH$_3$C$\underline{H_2}$O), 6.70 (1H, s, CH$_{isoxazole}$), 7.98 (2H, m, 2CH$_{ar}$), 8.16 (1H, d, J = 8.2, CH$_{ar}$). ^{13}C NMR (50 MHz, DMSO-d$_6$; δ, ppm): 13.88, 17.58, 19.68, 24.66, 52.03, 61.67, 102.00, 112.96, 18.41, 124.90, 125.74, 126.36, 129.34, 136.01, 146.71, 156.05, 159.42, 162.25, 164.73, 165.63, 167.85, 172.22, 174.40. **RCB22137**, Yield 82 %. M.p. 139-141 °C. MS (EI; rel. int., %): m/z 443 (M$^+$, 85). ^{1}H NMR (200 MHz, DMSO-d$_6$; δ, ppm): 1.76-2.03 and 2.04-2.31 (1H and 1H, m, CH$_2$C$\underline{H_2}$CH$_2$O), 2.44 (3H, s, CH$_3$), 2.74-3.21 (4H, m, C$\underline{H_2}$CH$_2$C$\underline{H_2}$O), 3.88 (s, 3H, COOCH$_3$), 6.71 (1H, s, CH$_{isoxazole}$), 7.98 (2H, m, 2CH$_{ar}$), 8.16 (1H, d, J = 9.1, CH$_{ar}$). ^{13}C NMR (DMSO-d$_6$; δ, ppm): 17.58, 19.67, 24.67, 30.74, 35.72, 102.02, 112.98, 118.41, 124.90, 125.74, 126.37, 129.34, 136.01, 146.69, 155.84, 159.90, 162.25, 164.73, 165.57, 167.85, 174.46.

Synthetic procedure for methyl ester of 5-(3-((2-methyl-4-(5-(trifluoromethyl)-1,2,4-oxadiazol-3-yl)phenyl)thio)propyl)isoxazole-3-carboxylic acid RCB22135

A mixture of **RCB18350** (0.39 g) and freshly prepared sodium methoxide (0.048 g) in methanol (8 mL) was stirred at room temperature for 2 days. After completion of the reaction, which was

monitored by TLC, the mixture was evaporated in vacuo, and the residue was recrystallized from methanol to afford **RCB22135** as a white solid. Yield 80 %. M.p. 118-120 °C. MS (EI; rel. int., %): m/z 427 (M^+, 91). ^{1}H NMR (200 MHz, DMSO-d_6; δ, ppm): 2.06 (2H, p, *J* = 7.5, $CH_2C\underline{H_2}CH_2O$), 2.34 (3H, s, CH_3), 3.03 (2H, t, *J* = 7.5, $C\underline{H_2}CH_2CH_2O$), 3.15 (2H, t, *J* = 7.2, CH_2O), 3.88 (3H, s, $COOCH_3$), 6.76 (1H, s, $CH_{isoxazole}$), 7.48 (1H, d, *J* = 9.0, CH_{ar}), 7.86 (2H, m, $2CH_{ar}$). ^{13}C NMR (50 MHz, DMSO-d_6; δ, ppm): 19.48, 25.01, 25.89, 29.70, 52.64, 101.99, 113.05, 118.47, 120.34, 125.37, 125.52, 128.13, 136.20, 141.90, 155.85, 159.94, 164.38, 168.19, 174.71.

Synthetic procedure for *N*-methyl-5-(3-((2-methyl-4-(5-(trifluoromethyl)-1,2,4-oxadiazol-3-yl)phenyl)thio)propyl)isoxazole-3-carboxamide RCB22167

A solution of RCB18350 (0.2 g) and methylamine 40 wt. % in H_2O (0.5 mL) in methanol (6 mL) was stirred at room temperature for 2 h. After completion of the reaction, which was monitored by TLC, the mixture was evaporated in vacuo, and the residue was recrystallized from ethanol to afford **RCB22167** as a white solid. Yield 94 %. M.p. 129-133 °C. MS (EI; rel. int., %): m/z 426 (M^+, 88). ^{1}H NMR (200 MHz, DMSO-d_6; δ, ppm): 2.04 (2H, p, *J* = 7.3, $CH_2C\underline{H_2}CH_2O$), 2.34 (3H, s, CH_3), 2.75 (3H, d, *J* = 4.6, $COONHC\underline{H_3}$), 3.00 (2H, t, *J* = 7.5, $C\underline{H_2}CH_2CH_2O$), 3.14 (2H, t, *J* = 7.2, CH_2O), 6.60 (1H, s, $CH_{isoxazole}$), 7.47 (1H, d, *J* = 8.9, CH_{ar}), 7.86 (2H, m, $2CH_{ar}$), 8.61 (1H, d, *J* = 4.6, $COON\underline{H}CH_3$). ^{13}C NMR (50 MHz, DMSO-d_6; δ, ppm): 19.74, 24.99, 25.72, 25.98, 29.68, 100.81, 107.60, 113.02, 118.45, 120.31, 123.88, 125.38, 128.12, 136.1, 141.92, 158.81, 159.00, 163.51, 164.39, 165.24, 166.12, 168.18, 173.70.

^{1}H and ^{13}C Spectra for Some Newly Synthesized Compounds

^{1}H NMR (200 MHz, DMSO-d$_6$) of **RCB22132**

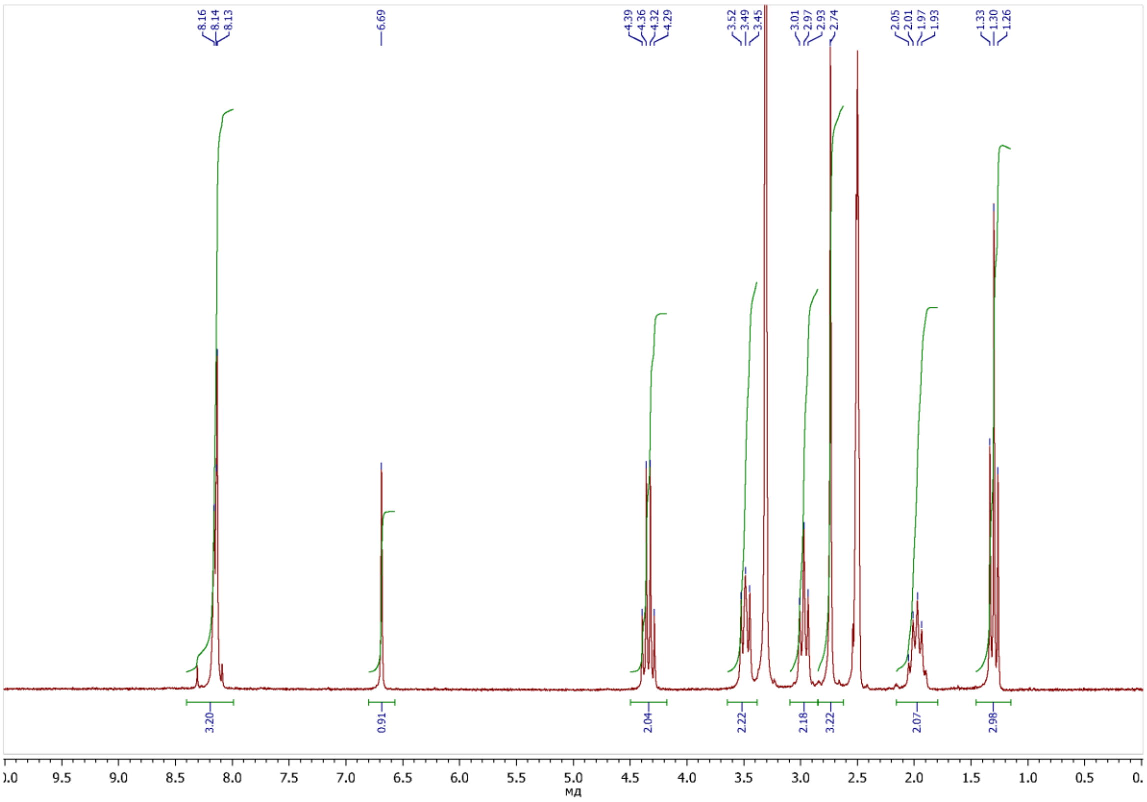

^{13}C NMR (50 MHz, DMSO-d$_6$) of **RCB22132**

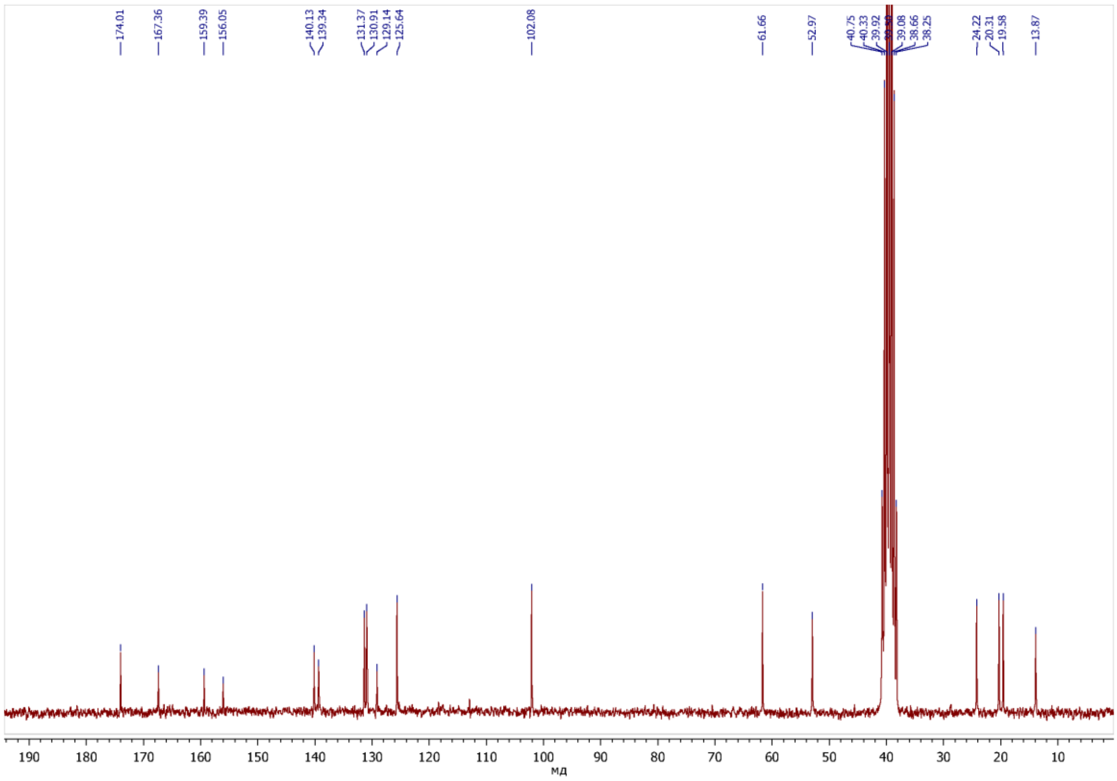

^{1}H NMR (200 MHz, DMSO-d_6) of **RCB22134**

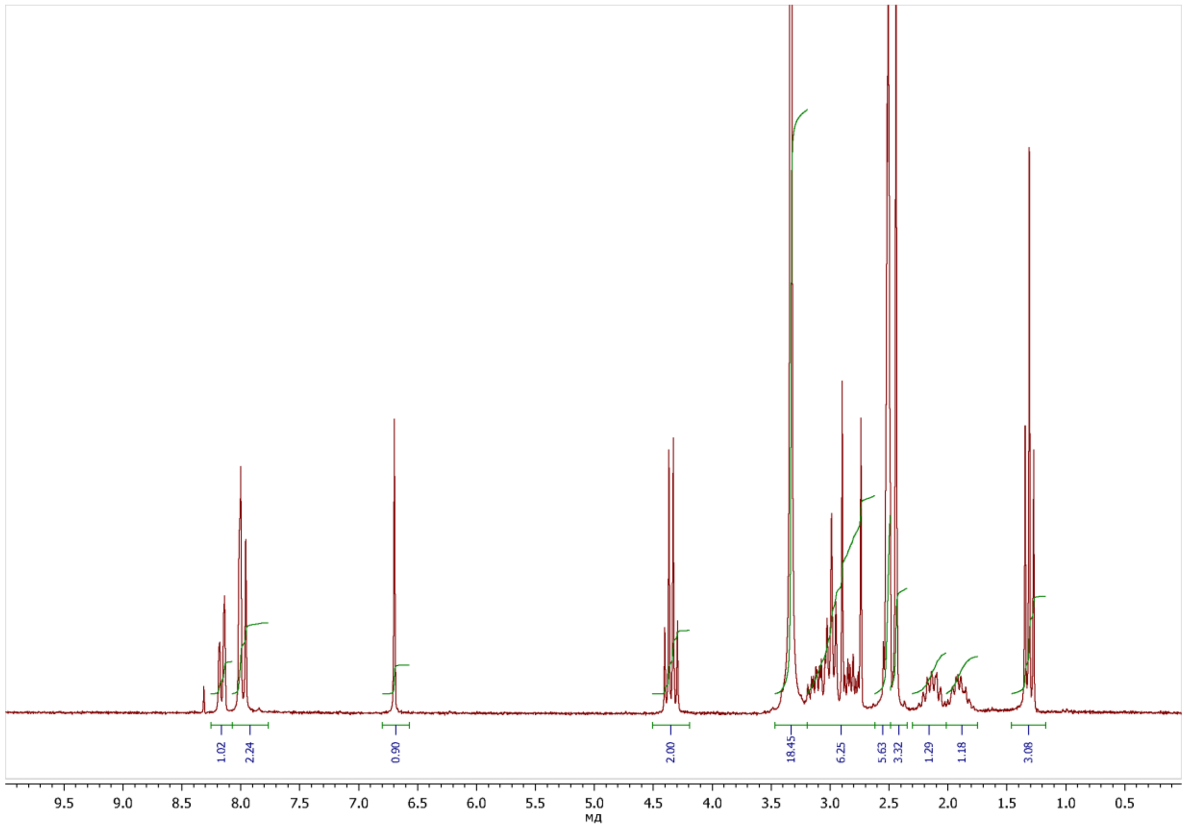

^{13}C NMR (50 MHz, DMSO-d_6) of **RCB22134**

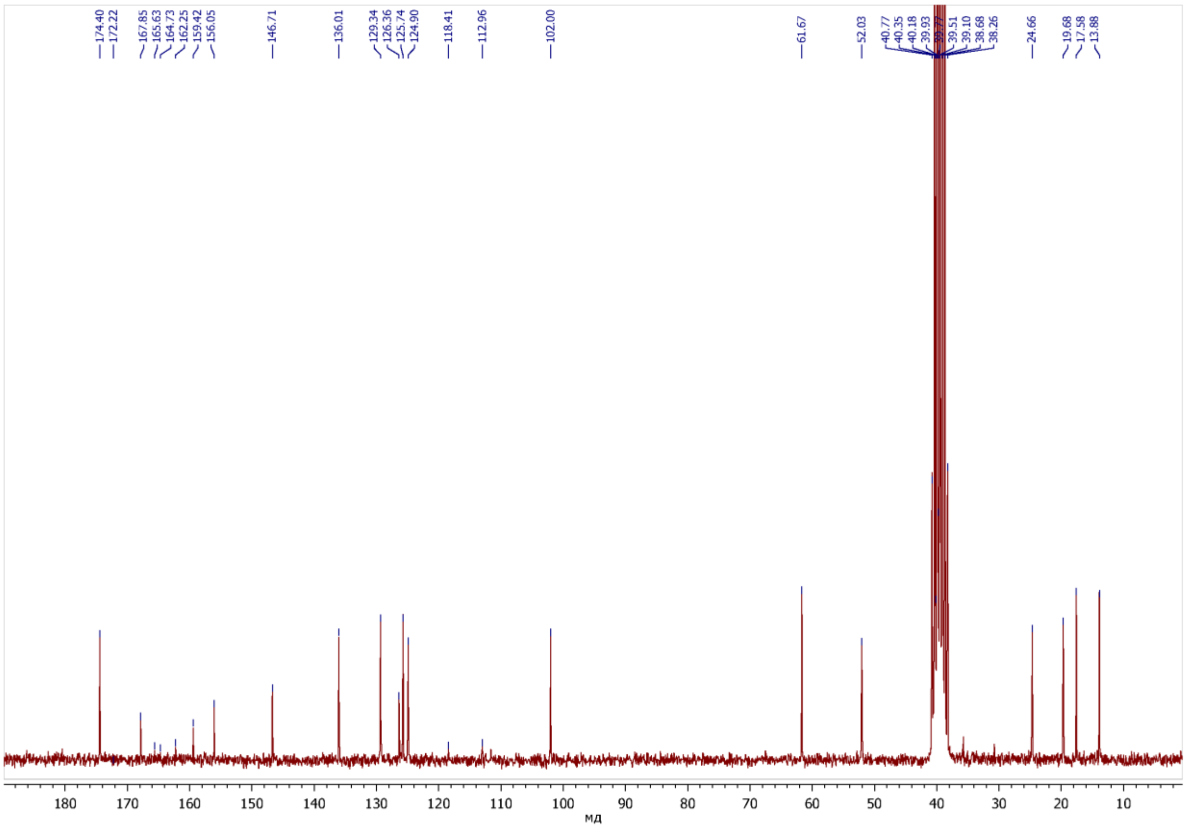

^{1}H NMR (200 MHz, DMSO-d$_6$) of **RCB22135**

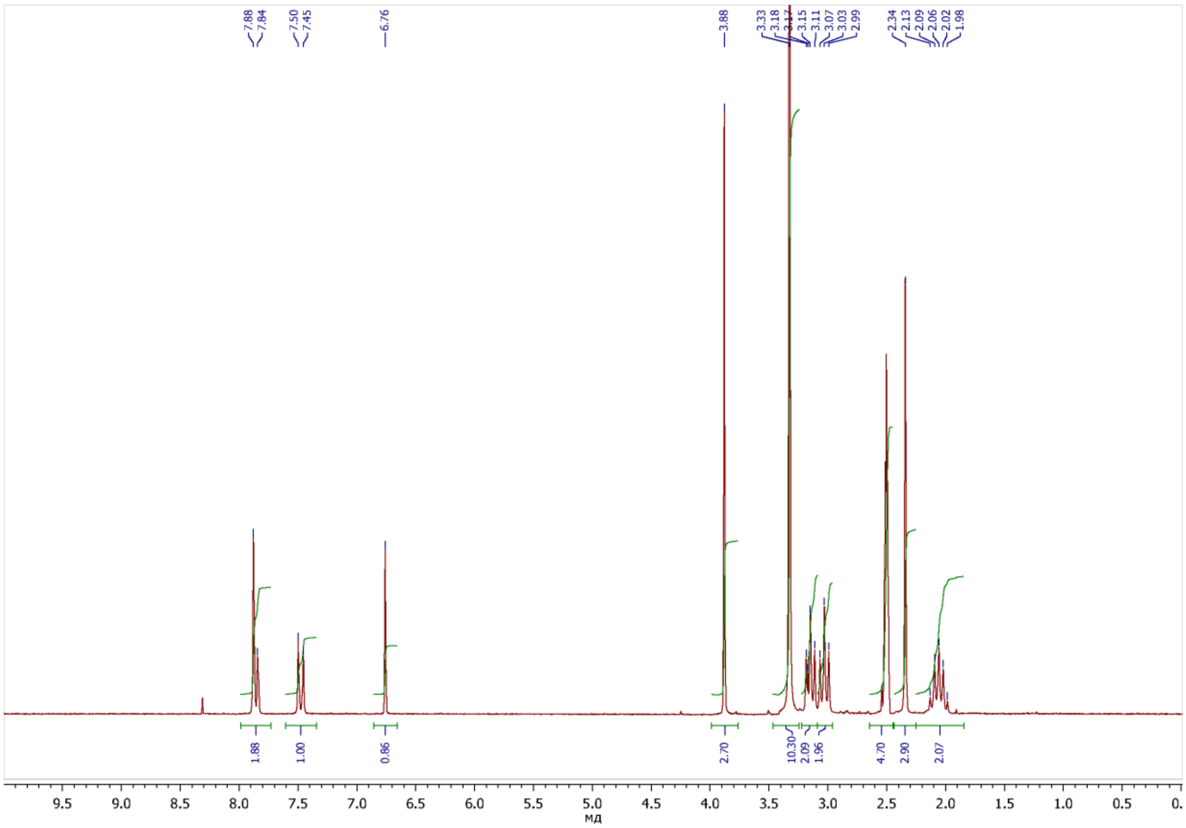

^{13}C NMR (50 MHz, DMSO-d$_6$) of **RCB22135**

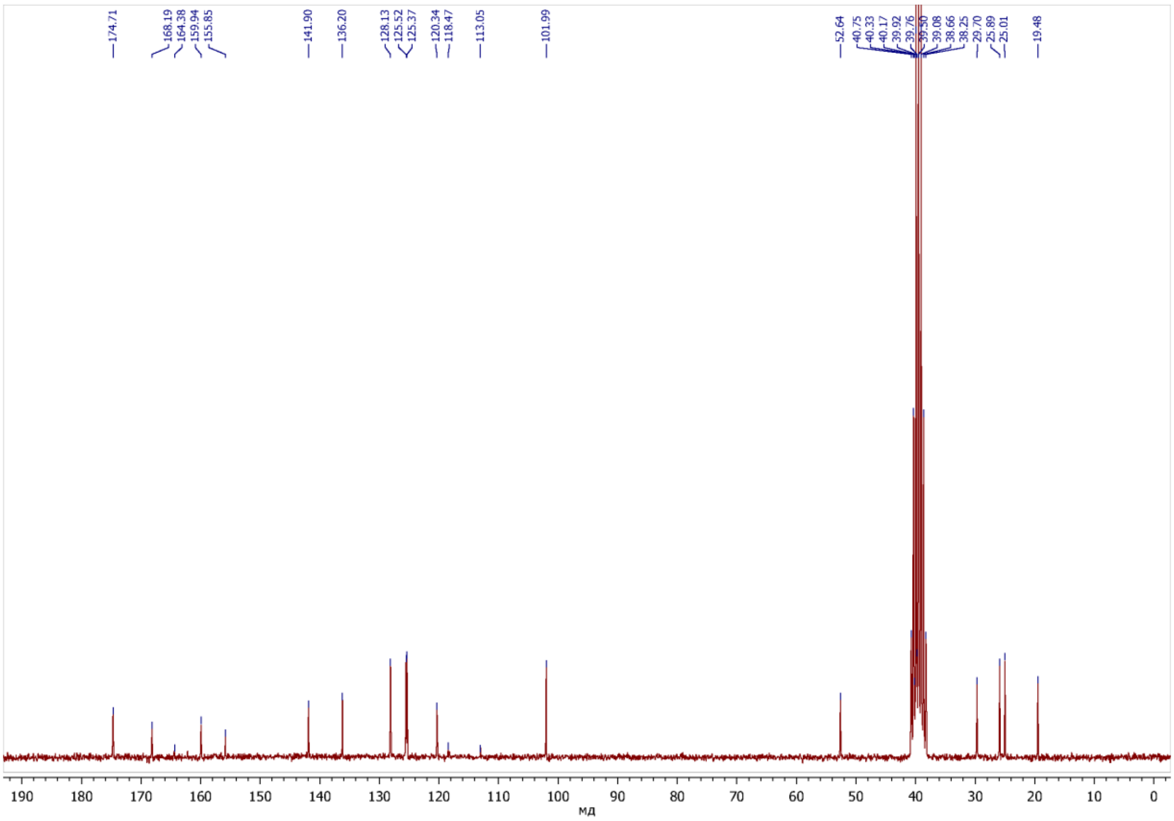

^{1}H NMR (200 MHz, DMSO-d_6) of **RCB22136**

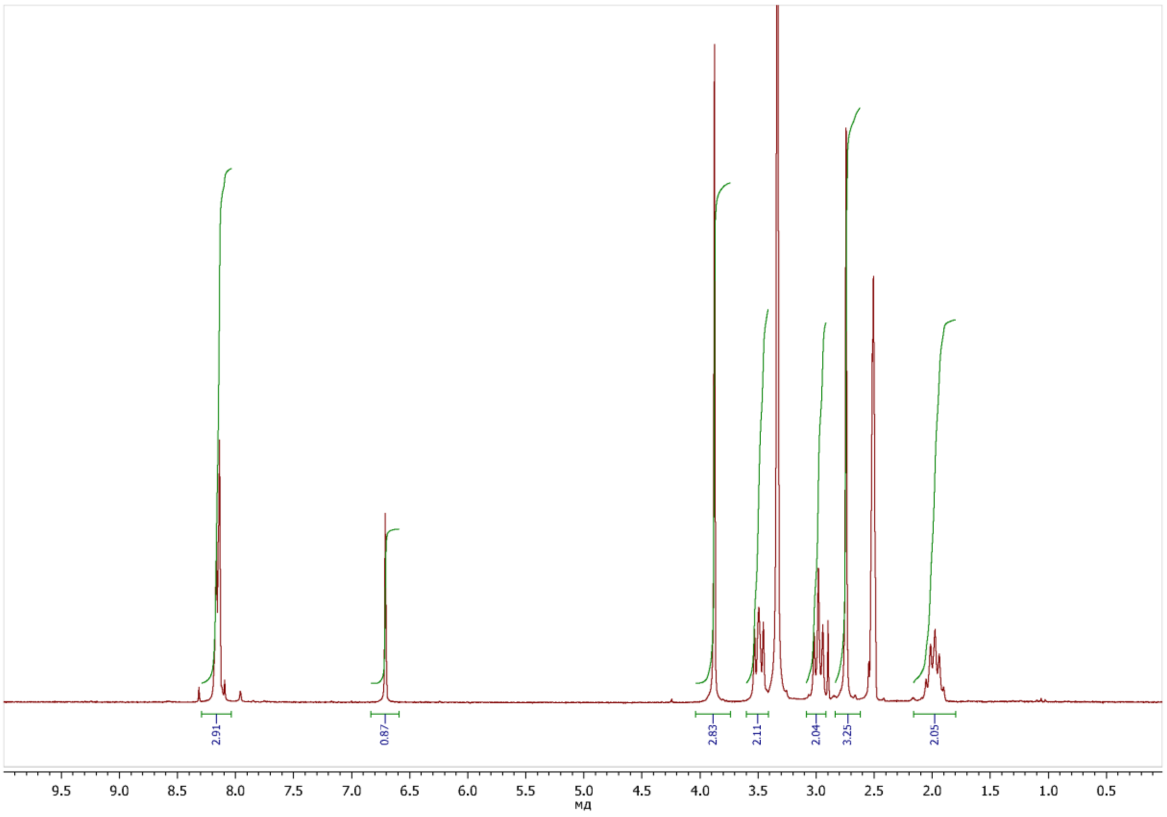

^{13}C NMR (50 MHz, DMSO-d_6) of **RCB22136**

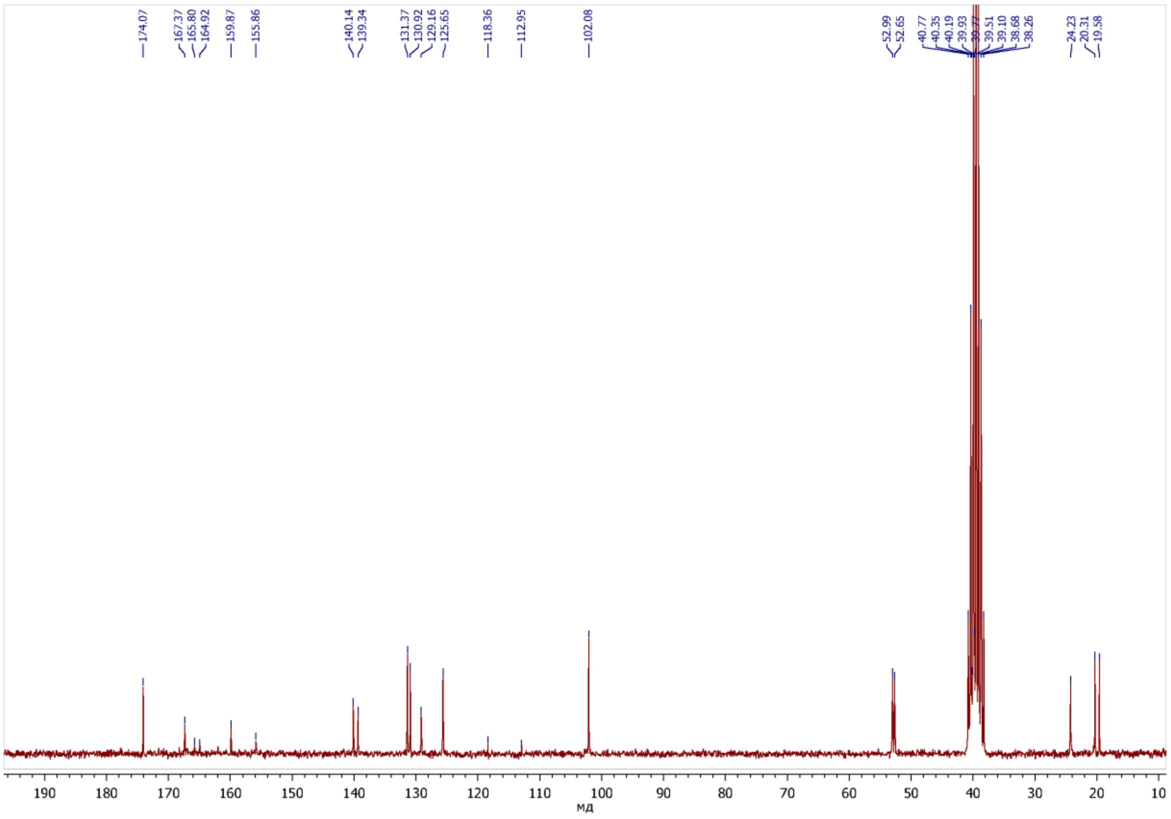

^{1}H NMR (200 MHz, DMSO-d_6) of **RCB22137**

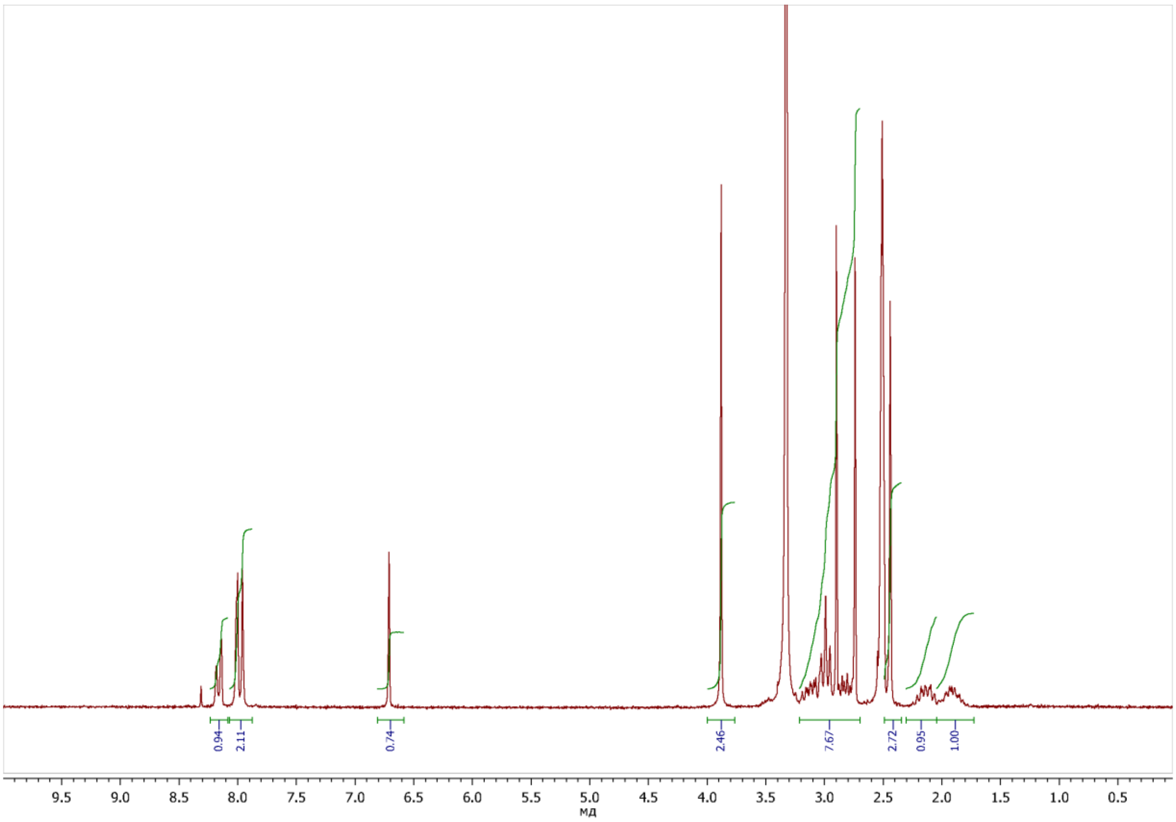

^{13}C NMR (50 MHz, DMSO-d_6) of **RCB22137**

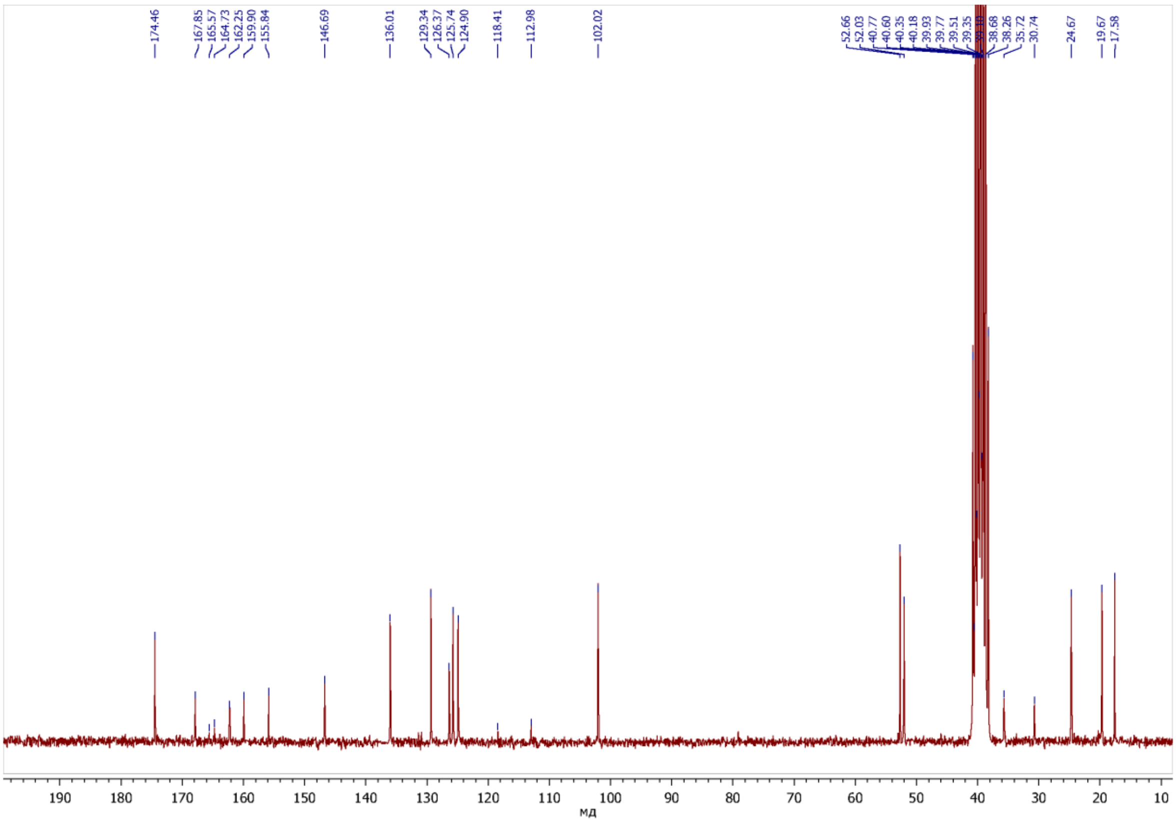

^{1}H NMR (200 MHz, DMSO-d_6) of **RCB22167**

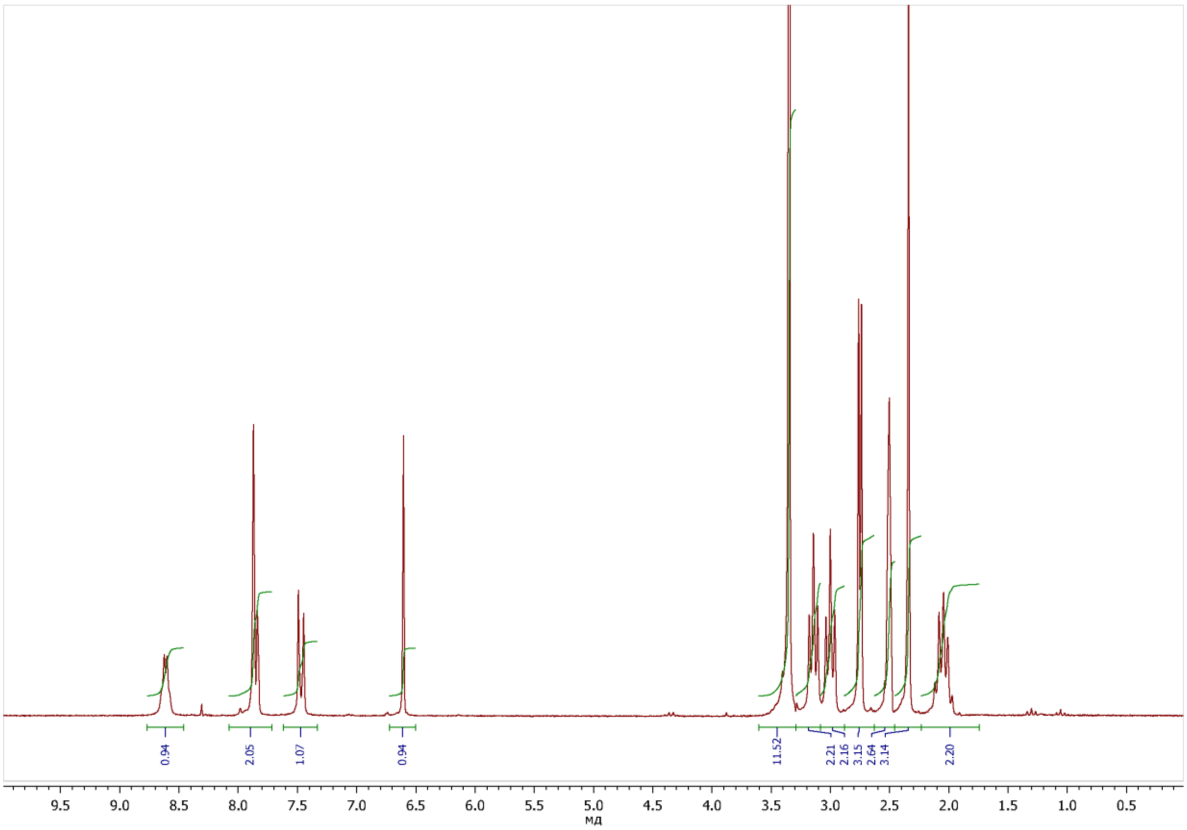

^{13}C NMR (50 MHz, DMSO-d_6) of **RCB22167**

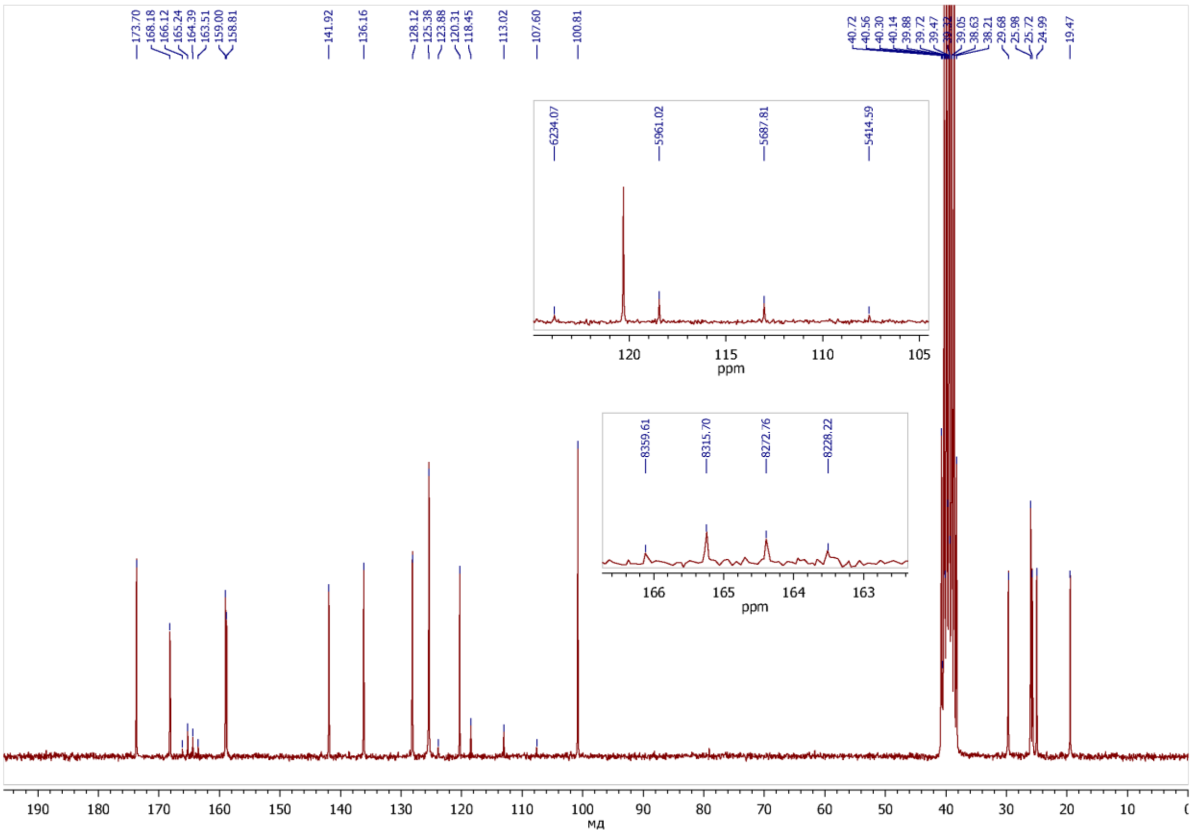

Supplement: Supplementary file 1 — id4c01030_si_001.pdf [file id4c01030_si_001.pdf]
